# Predicting adverse outcomes due to diabetes complications with machine learning using administrative health data

**DOI:** 10.1038/s41746-021-00394-8

**Published:** 2021-02-12

**Authors:** Mathieu Ravaut, Hamed Sadeghi, Kin Kwan Leung, Maksims Volkovs, Kathy Kornas, Vinyas Harish, Tristan Watson, Gary F. Lewis, Alanna Weisman, Tomi Poutanen, Laura Rosella

**Affiliations:** 1 Layer 6 AI, Toronto, ON Canada; 2 Department of Computer Science, University of Toronto, Toronto, ON Canada; 3 Dalla Lana School of Public Health, University of Toronto, Toronto, ON Canada; 4 MD/PhD Program, Temerty Faculty of Medicine, University of Toronto, Toronto, ON Canada; 5 ICES, Toronto, ON Canada; 6 Department of Medicine, Temerty Faculty of Medicine, University of Toronto, Toronto, ON Canada; 7 Department of Physiology, Temerty Faculty of Medicine, University of Toronto, Toronto, ON Canada; 8 Lunenfeld-Tanenbaum Research Institute, Mt. Sinai Hospital, Toronto, ON Canada; 9 Division of Endocrinology and Metabolism, Temerty Faculty of Medicine, University of Toronto, Toronto, ON Canada; 10 Vector Institute, Toronto, ON Canada; 11 Institute for Better Health, Trillium Health Partners, Mississauga, ON Canada; 12 Department of Laboratory Medicine & Pathology, Temerty Faculty of Medicine, University of Toronto, Toronto, ON Canada

**Keywords:** Health services, Epidemiology

## Abstract

Across jurisdictions, government and health insurance providers hold a large amount of data from patient interactions with the healthcare system. We aimed to develop a machine learning-based model for predicting adverse outcomes due to diabetes complications using administrative health data from the single-payer health system in Ontario, Canada. A Gradient Boosting Decision Tree model was trained on data from 1,029,366 patients, validated on 272,864 patients, and tested on 265,406 patients. Discrimination was assessed using the AUC statistic and calibration was assessed visually using calibration plots overall and across population subgroups. Our model predicting three-year risk of adverse outcomes due to diabetes complications (hyper/hypoglycemia, tissue infection, retinopathy, cardiovascular events, amputation) included 700 features from multiple diverse data sources and had strong discrimination (average test AUC = 77.7, range 77.7–77.9). Through the design and validation of a high-performance model to predict diabetes complications adverse outcomes at the population level, we demonstrate the potential of machine learning and administrative health data to inform health planning and healthcare resource allocation for diabetes management.

## Introduction

The global diabetes burden is projected to increase from 380 million people in 2013 to 590 million by 2035^1^. Patients living with diabetes have a higher risk for acute and long-term complications, such as hyperglycemia, nervous system damage, kidney disease, eye damage, and cardiovascular events, than the general population^2,3^. Furthermore, treatments for diabetes complications are a major contributor to the healthcare costs attributable to diabetes, particularly due to hospitalizations and emergency department visits^4,5^. Thus, predicting adverse outcomes due to diabetes complications is important for health system planning.

There is substantial evidence around the prevention of diabetes complications, as landmark studies have demonstrated the importance of controlling hyperglycemia, hypertension, hypercholesterolemia, and smoking cessation^6–10^. However, there are systems-level barriers, which compromise the ability to act upon this evidence and care for populations at scale^11^. These include socioeconomic status (SES) disparities broadly, shown internationally^12–14^, the high cost of medications^15,16^, access to care and healthcare personnel^17,18^, and the built environment^19,20^. Limitations in public health planning and healthcare resource allocation can contribute to “cascades in care” where those who are not receiving care will not meet the targets vital for complications prevention^21^.

**Fig. 1:**
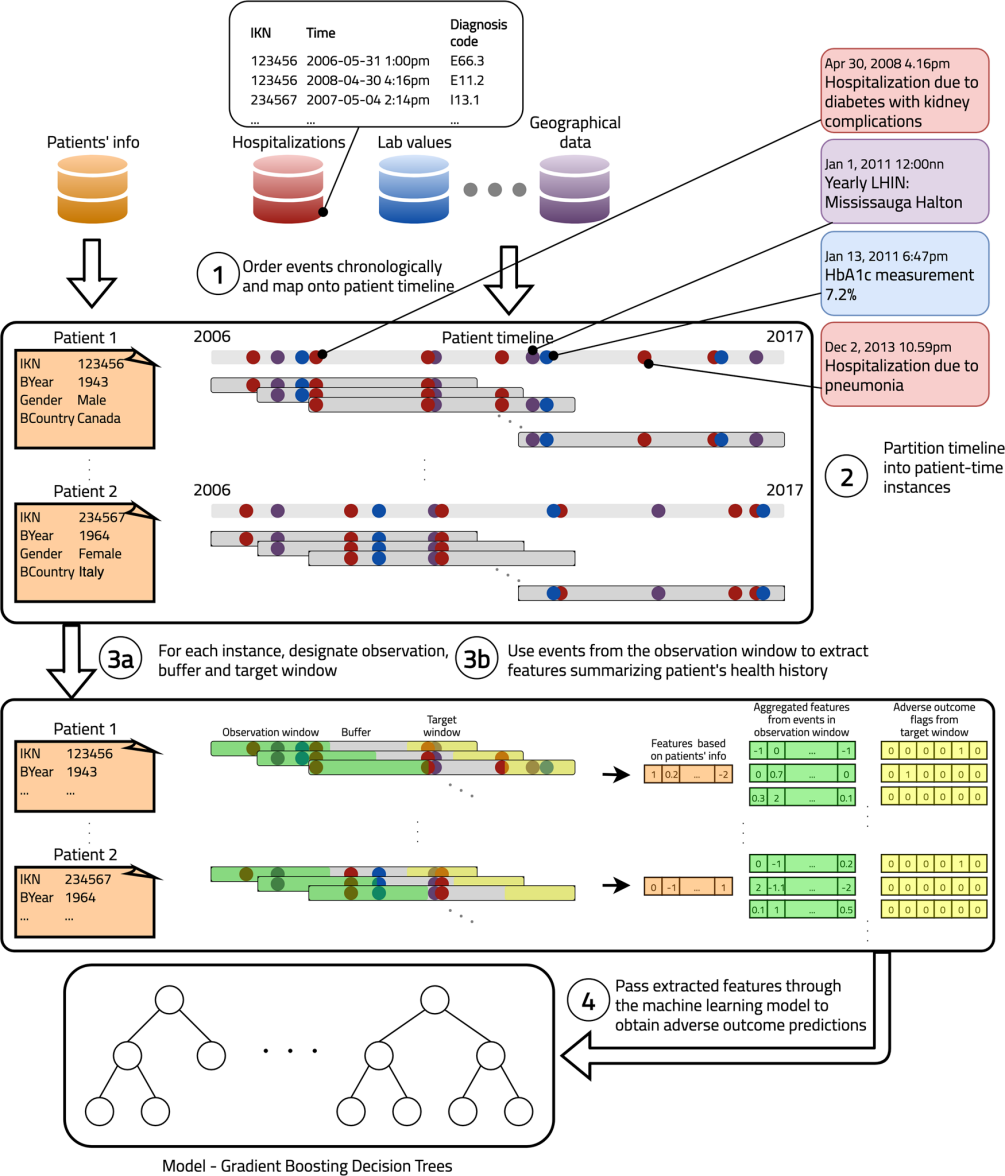
Overview of our end-to-end prediction pipeline. (Step 1) Events from multiple administrative health datasets are ordered chronologically and mapped onto patient timeline. (Steps 2 and 3a) Patient timeline is partitioned into patient-time instances and each instance is assigned an observation, buffer and target window. In this study, observation window is 2 years, buffer is 3 years, target window is 3 months and we use nonoverlapping target windows. To illustrate this partitioning, we consider the test period which runs from Jan 1, 2016 to Dec 31, 2016 as an example. In this period a given patient has four instances at Jan 1, 2016, Apr 1, 2016, Jul 1, 2016, and Oct 1, 2016. The first instance has observation window [Jan 1, 2011–Dec 31, 2012], buffer [Jan 1, 2013–Dec 31, 2015], and target window [Jan 1, 2016–Mar 31, 2016]. The second instance has observation window [Apr 1, 2011–Mar 31, 2013], buffer [Apr 1, 2013–Mar 31, 2016], and target window [Apr 1, 2016–Jun 30, 2016], and so on. (Step 3b) Events from the observation window are used to extract features that summarize patient’s health history up to the end of the observation window. (Step 4) Extracted features are passed to the machine learning model to generate adverse outcome predictions. The goal of the model is to accurately predict which instances will have an adverse outcome from each of the six complications in the target window.

Many prognostic models have been developed for diabetes complications in the clinical setting^22–24^, including more recent applications of machine learning approaches^25–34^. These models generally have made use of rich suites of features (e.g., body mass index, smoking status, biomarkers ranging from commonly ordered lipids to extensive genetic panels) extracted from electronic medical records (EMRs)^25,27,31–33^ or clinical trials^28,30^. However, while these models are important for clinical level risk prediction, they are not easily deployed by governments or private health insurance providers at the population level—which is precisely what is needed for addressing the aforementioned systemic barriers to diabetes complications care^35,36^. In contrast, administrative health data (AHD) consists of records collected automatically on diagnoses, procedures, medications, and demographics generated through the provision of health services by governments or other payers^37^. They most commonly do not contain imaging data, doctor text notes, laboratory results, or clinical measures. AHD are high-dimensional, and impossible to explore by clinicians or health systems planners manually. AHD have been long proposed as a tool to assess the quality of a healthcare system^38^, but they also represent an enabler for automated analytic approaches to drive the efficiency and effectiveness of primary and secondary health prevention efforts^39,40^. See Supplementary Table 6 for a more detailed comparison between EMRs and AHD. The purpose of this study is to develop a single, large-scale machine learning model for common adverse outcome prediction due to diabetes complications that can be applied on routinely collected AHD for the purposes of public health planning and healthcare resource allocation (Fig. 1). Adverse outcomes are the manifestation of complications in a manner that results in hospital or ambulatory care. It is not our goal for this model to be applied in the context of individual patient care. We base our study on the single-payer health insurance system in Ontario, Canada. Canada has established some of the most comprehensive administrative health data holdings in the world, covering nearly the total population, in part owing to its universal healthcare system^41^. To ensure broad patient coverage we apply minimal selection criteria, and only require patients to be alive and diagnosed with diabetes at some point in their life. We used a “2-claim” algorithm to flag diabetes in administrative health data^42^. Since this algorithm does not differentiate type 1 and type 2, the resulting cohort is made of patients diagnosed with both types^42^. This results in a large and diverse cohort of over 1.5 million patients with a broad representation of different socio-demographic groups and patterns of interaction with the healthcare system (e.g., frequency of doctor visits, availability of laboratory results etc.). Our results indicate that machine learning can be successfully leveraged to draw insights from administrative health data with minimal restriction, opening up avenues for the deployment of advanced population health management systems to improve health management, promote health equity, and reduce barriers to diabetes care with low per-patient overhead and cost.

## Results

### Cohort characteristics

We aim to predict three years in advance, whether a patient diagnosed with diabetes will experience a healthcare visit from an adverse outcome due to a diabetes complication within a target three month prediction window. For this study, an adverse outcome is defined as at least one hospitalization or ambulatory usage associated with any diabetes complication during the target prediction window. Note that our task differs from complication incidence prediction itself as multiple adverse outcomes can be associated with the same complication and can occur repeatedly as the complication evolves. Adverse outcomes generally indicate significant negative events during a patient’s diabetes progression, and can be both detrimental to quality of life and place considerable cost burden on the healthcare system. Accurate advanced prediction can support preventative measures, and aid with how resources are deployed and managed within a health system. We target the following five complications: severe hyper/hypoglycemia, tissue infection, retinopathy, cardiovascular events (e.g., stroke), and amputation. Specific definitions for each complication’s adverse outcomes are provided in the Supplementary Table 5. This set covers most major complications, with the exception of those that have very limited data in our cohort such as kidney failure or erectile dysfunction.

After applying the selection criteria, we obtain a cohort of 1,567,636 patients. The model is trained using the data from 1,029,366 patients, then validated and tested on the distinct sets of 272,864 and 265,406 patients, respectively. Patients in all sets are selected at random. Both validation and testing are done forward in time so all evaluation is out-of-time and out-of-sample. Given the large-scale size of our final cohort, we do not use k-fold cross-validation and stick to a single, fixed validation set, as is common practice with very large datasets^43,44^. We use patient-time instances for all modeling, where each instance represents a view of the patient at a specific point in time. All patients thus have multiple associated instances as we slide the model across time. This simulates the real-life application of advanced population risk assessment systems, that are typically used to continuously monitor patients at regular time intervals. Cohort statistics are summarized in Table 1. We observe that instance incidence rates within the narrow three month window varies significantly across complications between 0.04% (retinopathy) and 1.08% (cardiovascular events). In all cases, the binary classification is severely imbalanced.

**Table 1:** Patient and instance counts across complications and population subgroups.

	Training		Validation		Test	
	(Jan. 2011–Dec. 2014)		(Jan. 2015–Dec. 2015)		(Jan. 2016–Dec. 2016)	
	Patients	Instances	Patients	Instances	Patients	Instances
*Full cohort*	1,029,366	15,862,818	272,864	1,077,964	265,406	1,046,122
Adverse outcomes^a^						
Hyper/Hypoglycemia	12,015 (1.17%)	16,462 (0.10%)	1116 (0.41%)	1279 (0.12%)	1191 (0.45%)	1396 (0.13%)
Tissue infection	67,200 (6.53%)	92,089 (0.58%)	5946 (2.18%)	6782 (0.63%)	6047 (2.28%)	6898 (0.66%)
Retinopathy	4875 (0.47%)	5902 (0.04%)	429 (0.16%)	482 (0.05%)	386 (0.15%)	418 (0.04%)
Cardiovascular events	105,310 (10.23%)	160,084 (1.01%)	10,030 (3.68%)	12,102 (1.12%)	9456 (3.56%)	11,254 (1.08%)
Amputation	44,560 (4.33%)	54,947 (0.35%)	3552 (1.30%)	3760 (0.35%)	3479 (1.31%)	3728 (0.36%)
Sex						
Male	535,903 (52.06%)	8,250,936 (52.01%)	142,037 (52.05%)	560,863 (52.03%)	137,315 (51.74%)	540,721 (51.69%)
Female	493,463 (47.94%)	7,611,882 (47.99%)	130,827 (47.95%)	517,101 (47.97%)	128,091 (48.26%)	505,401 (48.31%)
Age group^b^						
<20	17,197 (1.67%)	235,540 (1.48%)	3304 (1.21%)	13,160 (1.22%)	2873 (1.08%)	11,381 (1.09%)
20–44	187,359 (18.20%)	2,495,146 (15.73%)	35,783 (13.11%)	142,001 (13.17%)	32,683 (12.31%)	129,044 (12.34%)
45–64	533,188 (51.80%)	7,439,656 (46.90%)	123,393 (45.22%)	490,343 (45.49%)	117,955 (44.44%)	467,611 (44.70%)
65–79	345,604 (33.57%)	4,434,449 (27.96%)	84,375 (30.92%)	332,809 (30.87%)	85,225 (32.11%)	336,113 (32.13%)
80+	108,778 (10.57%)	1,258,027 (7.93%)	26,009 (9.53%)	99,651 (9.24%)	26,670 (10.05%)	101,973 (9.75%)
Immigration status^c^						
Immigrant	184,109 (17.89%)	2,770,495 (17.47%)	51,432 (18.85%)	202,624 (18.80%)	51,488 (19.40%)	202,458 (19.35%)
Long-term resident	845,257 (82.11%)	13,092,323 (82.53%)	221,432 (81.15%)	875,340 (81.20%)	213,918 (80.60%)	843,664 (80.65%)
Material deprivation marginalization score^d^						
1st quintile	180,587 (17.54%)	2,794,415 (17.62%)	48,768 (17.87%)	192,798 (17.89%)	47,502 (17.90%)	187,511 (17.92%)
2nd quintile	189,937 (18.45%)	2,935,301 (18.50%)	50,631 (18.56%)	200,175 (18.57%)	49,734 (18.74%)	196,204 (18.76%)
3rd quintile	199,671 (19.40%)	3,078,263 (19.41%)	52,727 (19.32%)	208,256 (19.31%)	51,794 (19.52%)	204,184 (19.52%)
4th quintile	207,273 (20.14%)	3,190,758 (20.11%)	54,447 (19.95%)	215,020 (19.95%)	52,419 (19.75%)	206,503 (19.74%)
5th quintile	229,932 (22.34%)	3,527,905 (22.24%)	60,433 (22.15%)	238,595 (22.13%)	58,477 (22.03%)	230,181 (22.00%)
Ethnicity marginalization score^d^						
1st quintile	170,001 (16.52%)	2,617,658 (16.50%)	43,868 (16.08%)	173,045 (16.05%)	41,958 (15.81%)	165,122 (15.78%)
2nd quintile	167,063 (16.23%)	2,579,779 (16.26%)	43,594 (15.98%)	172,093 (15.96%)	41,982 (15.82%)	165,391 (15.81%)
3rd quintile	168,147 (16.34%)	2,598,752 (16.38%)	44,389 (16.27%)	175,483 (16.28%)	42,785 (16.12%)	168,682 (16.12%)
4th quintile	192,984 (18.75%)	2,984,352 (18.81%)	51,214 (18.77%)	202,523 (18.79%)	50,409 (18.99%)	198,977 (19.02%)
5th quintile	309,205 (30.04%)	4,746,101 (29.92%)	83,941 (30.76%)	331,700 (30.77%)	82,792 (31.19%)	326,411 (31.20%)

We breakdown the cohort into age, sex, immigration status, material deprivation marginalization, and ethnicity marginalization. The observation window, buffer and target window are 2 years, 3 years, and 3 months, respectively. Target window date ranges are shown in brackets for training, validation, and test sets.

^a^A patient is considered to have an adverse outcome if there is a corresponding event anywhere in the training, validation, or test period. Similarly, an instance is considered to have an adverse outcome if there is a corresponding event in its target window.

^b^Age is computed at the start of training, validation, and test periods for each patient, and at the start of the observation window for each instance.

^c^Long-term residents correspond to patients born in Canada or who immigrated to Canada before 1985. Our immigrants cohort contains patients born in 19 different countries, from diverse regions such as South Asia, North Africa, and Eastern Europe. See Supplementary Material Table 1 for the details of the number of immigrants born in each country.

^d^Ethnicity and deprivation marginalization scores quantify the degree of marginalization within each District Administration (DA) according to ethnic concentration and material deprivation. A DA typically encompasses a few hundred inhabitants. These two scores are quintiles ranging from 1 to 5 based on each patient’s history from the 2004–2008 period, where five represents a highest degree of marginalization.

For each patient, we consider 11 years of history from January 2006 to December 2016, and aggregate data from multiple administrative health sources such as demographics, outpatient doctor visits, hospitalizations, laboratory tests, etc. Figure 1 outlines our end-to-end pipeline. We first order all events from each source chronologically, and partition this data into patient-time instances. For each instance we then extract features that summarize a patient’s health history at that point in time, and pass them to our machine learning model to get adverse outcome predictions.

### Model performance

**Fig. 2:**
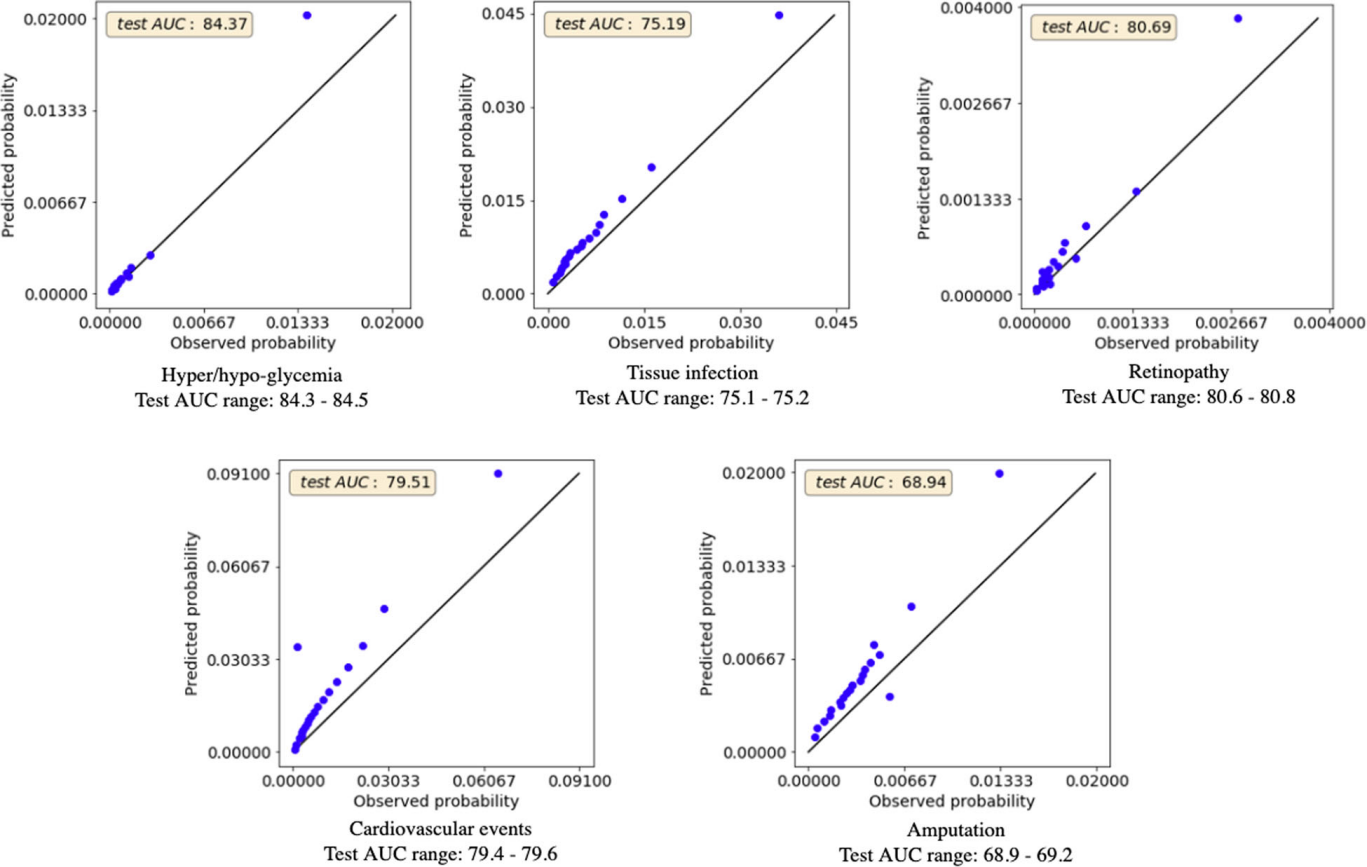
Test set AUC and calibration curves for all six complications. The average test AUC is 77.74 (77.7–77.9). The model is retrained five times with random restarts. The reported AUC results are averaged across the restarts. Corresponding ranges are also shown, low variance signifies that the results are stable. Calibration curves are computed using 20 bins of identical size, well-calibrated models have curves close to the identity line. Incidence rates vary significantly across complications (from 0.04 to 1.01%) so calibration curves have correspondingly different ranges.

Figure 2 shows Area Under the Receiver Operating Curve (AUC) for predictive performance as well as calibration curves for each complication. We compute the AUC using all instances from the test cohort to measure model performance for the entire test time period from January to December 2016. Given that the incidence rates vary significantly across complications, the calibration curves have correspondingly different ranges. Furthermore, since all incidence rates are very low, we do not compute the Brier score as it was shown to be not well suited for rare events^45^. Calibration curves are computed using 20 bins of identical size, and we plot “observed” and “predicted” probabilities for each bin. We retrain the model five times with random restarts, and report AUC ranges across the restarts. Our model achieves an average AUC of 77.74 (77.7–77.9) over the test set. The best and worst AUCs of 84.4 (84.3–84.5) and 68.9 (68.9–69.2) are obtained for adverse outcomes due to hyper/hypoglycemia and amputation, respectively.

### Feature contribution

**Fig. 3:**
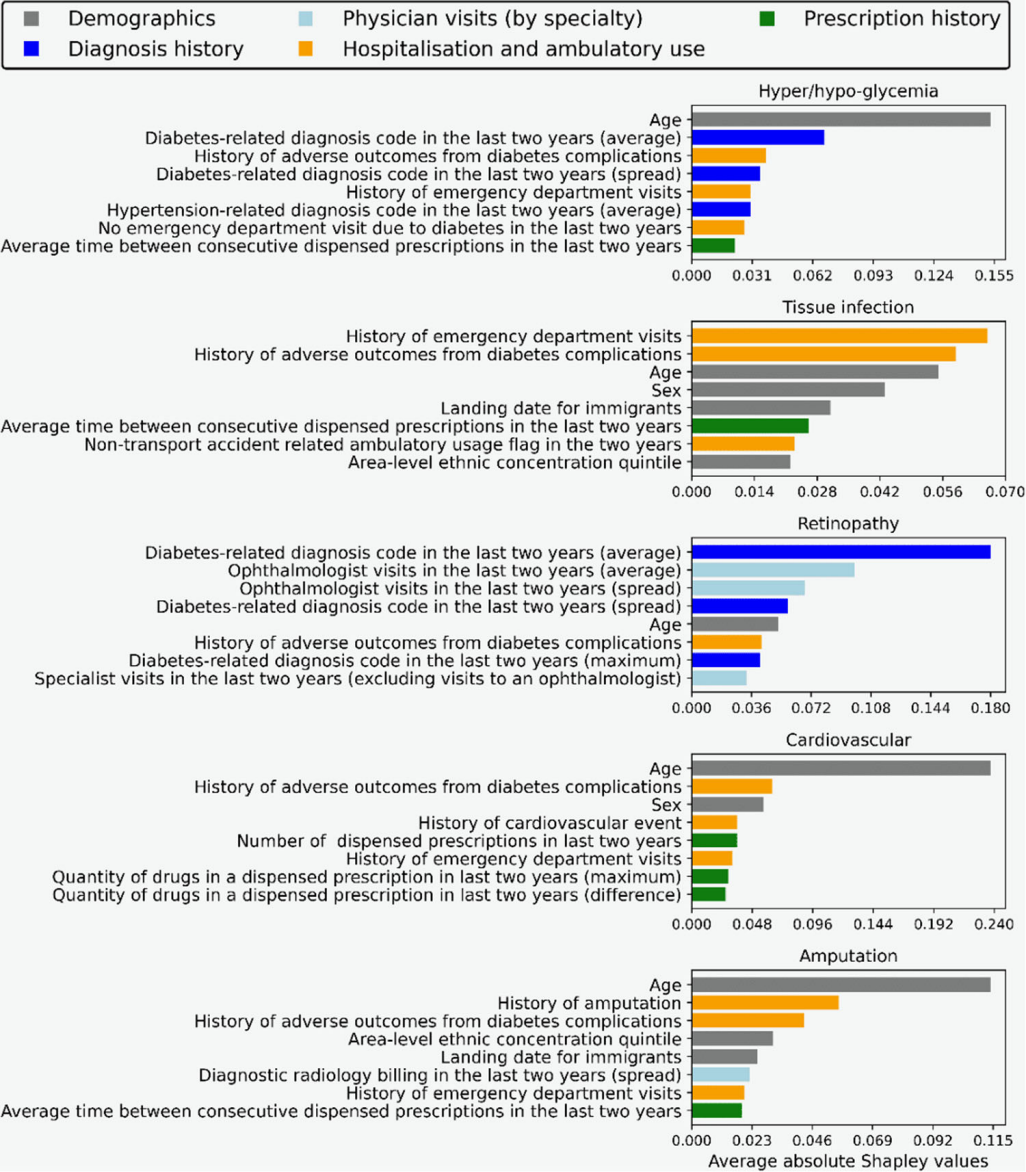
Top eight features for each complication. For each feature we show the corresponding administrative health dataset where it is derived from, and the magnitude of the contribution to the model. The contribution is measured using the mean absolute Shapley values (see Methods) over a large random sample of 10,000 test instances. The feature contributions here do not represent causal effects, and only indicate correlation with the target predicted adverse outcomes as captured by the model.

Figure 3 displays features that contribute the most to model prediction as determined by the mean absolute Shapley values (see Methods for further details)^46^. For each complication we show the top eight most predictive features, and the corresponding administrative health dataset where each feature is derived from. We observe that across all complications the model is leveraging features from multiple datasets to generate predictions. This can be partially attributed to the fact that each individual dataset is sparse, and thus cannot be used exclusively even if it is highly predictive. We also observe that while there are some commonalities, such as the age that is used for all complications, most top features differ for each complication. For instance, diagnosis history is important for hyper/hypoglycemia and retinopathy, while it is absent from the top eight features for the other three complications.

### Cost analysis

**Fig. 4:**
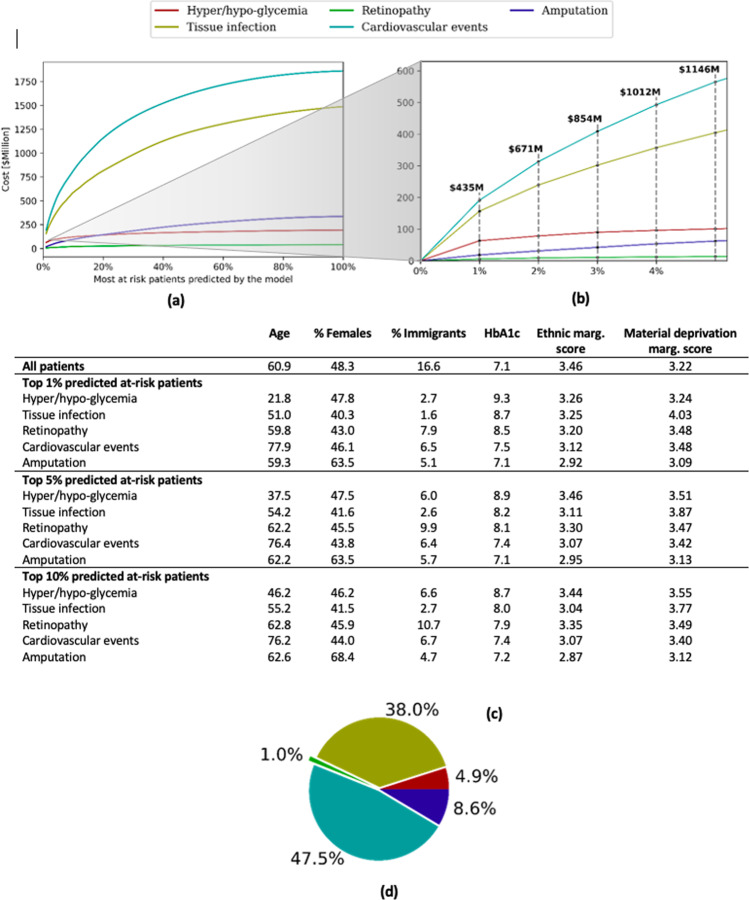
Adverse outcome cost analysis and high-risk statistics across complications. The total annual cost for adverse outcomes across all five complications is estimated to be ~$3.9B. **a** Annual cost for most at-risk patients predicted by our model. For each complication, we sort all patients by the predicted likelihood of adverse outcome, then compute total cost for each percentage of patients in the sorted list from top 1–100%. **b** A detailed sub-view from panel **a** for top 5% of predicted patients with the total cost across all six complications. **c** Statistics for the top 1% of most at risk patients predicted by our model. We analyze age, sex, immigration status, and HbA1c values, and compare them to the full cohort. All statistics are computed at the end of the observation window for each patient when the model makes its prediction. **d** Breakdown of the contribution to the total annual cost by complication.

Due to the high incidence rate and significant number of complications, diabetes imposes one of the heaviest cost burdens on the healthcare system^47,48^. In Ontario, Canada with a population of 14.5 million people in 2019, ambulatory use and hospitalizations due to the diabetes complications considered here alone cost over $3.9 billion per year. This estimate is obtained using the validated costing methodology developed by Wodchis et al.^49^ and we analyze cost further in Fig. 4. The costing algorithm gives us the annual patient expenditure for each patient and each healthcare channel (hospitalizations, ambulatory usage, drugs, etc). Since we also have access to each patient’s health history, we computed estimations of the cost of each adverse outcome. See Methods for more details on the cost computation.

Figure 4a–c is built by ranking patients by decreasing likelihood of getting an adverse outcome as predicted by the model. Figure 4d shows the proportion of the total cost that is contributed by each of the five complications. Adverse outcomes due to cardiovascular events and tissue infection complications account for the largest proportion of the total cost, jointly contributing over 82%. This is expected since these are the most frequent complications in our cohort with 3.56% and 2.28% test patient incidence rate, respectively.

Figure 4a shows the total annual cost for most at risk patients predicted by our model. For each of the five complications, we sort patients according to the predicted likelihood of adverse outcome, then compute the annual cost for each percentage of patients in this sorted list from top 1–100%. As seen in the figure, the model captures around $440 M (11.1%), $850 M (21.8%), and $1.15B (28.1%) of the total annual cost in the top 1%, 3%, and 5% of predicted patients, respectively. Figure 4c further summarizes statistics for the top 1% of the most at-risk patients predicted by the model. We observe several interesting patterns. First, adverse events due to hyper/hypoglycemia are typically predicted for much younger patients with an average age of 21.8 years old compared to 60.9 for the full cohort. Second, the ratio of female versus male patients remains roughly constant across complications except for amputation where the fraction of female patients is higher. Third, the proportion of most at-risk patients that are immigrants is significantly lower for all complications relative to the full cohort. The top 1% most at-risk patients similarly have a lower ethnicity marginalization score for all complications. Finally, HbA1c measurements are higher for patients predicted for hyper/hypoglycemia than other complications. It has been shown that patients with diabetes with an out-of-control glycemia are at higher risk of severe hypoglycemia^50^. We emphasize that these findings in Fig. 4c may not fully be relevant in a clinical setting, but reflect attributes of the training data.

## Discussion

This research demonstrated the feasibility of applying machine learning methods to administrative health data for public health planning. Our model can predict the 3-year risk of adverse outcomes due to diabetes complications (hyper/hypoglycemia, tissue infection, retinopathy, cardiovascular events, and amputation) with a test AUC of 77.7 (range 77.7–77.9, Fig. 2). It was not our goal for this model to be used for individual level patient care. Our model was trained on data from over 1.5 million patients from Ontario, which is among one of the most diverse populations in the world and, to our knowledge, one of the largest prediction modelling studies that takes into account multiple types of diabetes complications^22–34,51–53^. Our model was also well-calibrated and showed good discrimination.

While diabetes complications have been better managed in recent years, they remain a large burden because the incidence of diabetes continues to grow and even in the presence of interventions, not all cases can be prevented^54^. Thus, there is a need to effectively manage diabetes complications at both the individual patient and system levels. This is further emphasized as increasing age and years lived with diabetes have been found to independently predict diabetes morbidity and mortality^55^. Moreover, it has been well established that the complications of diabetes drive costs^4,5^. In Ontario alone, with a population of 14.5 million in 2019, adverse outcomes due to diabetes complications had an annual cost of over $3.9 billion, making diabetes a critical condition that warrants investment into analytic data-driven solutions for health system planning.

Health systems planning, for diabetes and other conditions, requires accurate assessments of population risk^35,36^. From the cost analysis in Fig. 4b, we observe that the top 1% of most at-risk patients predicted by our model account for over $440 M or 11% of the overall annual cost. This increases to over $850 M or nearly 22% of the top 3% of patients’ total cost. In contrast, random selection would only capture 1% and 3% of the cost, respectively. Targeting policy interventions (e.g., subsidizing access to fruits and vegetables, community planning to facilitate active transportation) and resource allocation (e.g., incentivizing physicians to have more intensive diabetes follow-up care, either virtually or in-person) to communities projected at highest risk based on our model outputs could help maximize their effectiveness in changing the trajectories of diabetes complications^11,36^.

The observed differences in model calibration across complication type may be impacted by the inclusion of both episodic and progressive types of diabetes complications, which by nature have a different epidemiology and trajectory^2^. More specifically, episodic complications, such as tissue infection, can be treated and recur multiple times, whereas progressive complications, such as cardiovascular disease, generally result over an elongated period of time due to chronic damage to the organ system^2^. The overprediction of high-risk individuals could also be due to the relationship between age and years lived with diabetes as key drivers of complications^55^. Finally, it is possible that our overprediction of those at high risk could be due to the lack of valuable clinical features such as body mass index, smoking status, biomarkers in AHD. At the population level, applications that overpredict would still be appropriate for targeting resources and identifying individuals that would benefit from closer follow-up, including the use of other prediction models which include biomarkers and other individual risk factors.

The analysis of top features in Fig. 3 provides insight into the types of information used by our model to make predictions for each complication. Explainability is a major benefit of decision tree models, and is one of the main reasons why we focus on decision trees for this study. Administrative health databases typically have billions of records spread across multiple datasets making it highly challenging to work with. Moreover, predictive patterns inferred by the model at this scale can identify new trends at the population level (or validate existing hypotheses)^56^. In Fig. 3, we observe that socio-demographic factors such as length of stay in Canada for immigrants and ethnic concentration in the area of residence, play an important role in model prediction. We consistently found that features based on immigration status, age/sex, area of residence (particularly census statistics such as neighbourhood-level income, unemployment, ethnic concentration etc.) and other related information appeared within the top 20 most predictive features. This is also observed from Fig. 4d, which shows that there are significantly fewer immigrants and lower ethnicity marginalization in the top 1% of the most at-risk patients predicted by the model as compared to the full cohort. Lower proportion of immigrants aligns with previous studies showing that immigrants have a different diabetes trajectory and are less at risk for these complications^57^. Clinical prediction models generally exclude such types of features and mainly focus on health data for each patient. Our results indicate that the social determinants of health, even at the census level, can be highly predictive for severe health outcomes. Thus the application of a model such as ours for population health planning, which leverages detailed information on the social determinants to allocate resources and plan policies to improve diabetes complications outcomes could offer a data-driven approach to addressing health disparities^58–60^.

Our study features a number of important strengths and contributions. The proposed model was developed and tested on a large cohort of over 1.5 million patients with minimal exclusion criteria, capturing virtually all incidences of target adverse outcomes. The cohort is ethnically diverse, with wide representation from across world regions. We demonstrate the applicability of machine learning methods using population data available in multiple jurisdictions around the world. We conducted extensive feature engineering and selection to capture correlations between different AHD sources and target outcomes. The final model has over 700 features from a variety of datasets such as demographics, census information, laboratory results, diagnosis history, physician billing claims, hospitalization and ambulatory usage, prescription medication history and others. Given the nature of administrative data, we believe that our approach could be applied for the forecasting of other chronic diseases at the population level. This is especially important given rising rates of multimorbidity internationally. One study in 2009 found that 24.3% of Ontarians were diagnosed with multiple comorbidities^61^. Since AHD is thought to be the most basic level of information collected by a healthcare system, we believe that our approach to population-level risk prediction would be feasible in other jurisdictions with universal health coverage and databases suitable for linkage such as the Scandinavian countries, United Kingdom, Australia, and New Zealand or within large private insurers in the United States. Finally, modern machine learning approaches are often criticized for lack of interpretability, and are sometimes referred to as black-box models^62^. Using a model based on decision trees enabled us to determine which features are important for prediction, and how they are combined inside the model. This is important for transparency and practical deployment of such systems that clinical and health system specialists need to be audited.

Despite the mentioned strengths, our study also has several important limitations. First, we are limited by the algorithm that we used to flag diabetes and build our cohort^42^. This “2-claim” algorithm has a specificity of 97%, meaning that there are almost no healthy patients in our cohort. However, its 86% sensitivity means that we did not capture all patients diagnosed with diabetes. Moreover, we are working with a joint cohort of patients diagnosed with type 1 and patients diagnosed with type 2. While patients diagnosed with both types share the complications and adverse outcomes we explored in this paper, their diabetes trajectory differs, with type 1 patients typically being diagnosed at a much younger age^2^. We considered using a validated type 1 diabetes algorithm^63^ to identify and remove the type 1 subcohort, but with a sensitivity of 80.6% on administrative health data, it would leave out hundreds of patients diagnosed with type 1 in our cohort. We argue that it is preferable for a system-level analysis to predict adverse outcomes of diabetes complications from both patients diagnosed with type 1 and patients diagnosed with type 2 since systems-level barriers are shared between the two populations^11^. We focused on hospitalization and ambulatory care service usage due to diabetes complications. Hence, we do not account for associated adverse events treated in the primary care settings as they could not be identified accurately in our data. In addition, we lacked prescription information for individuals under 65 years old (Ontario’s health system provides age-based drug coverage for individuals 65 years and older and those receiving social assistance). If available, they may improve predictive performance even further. More generally, as AHD systems around the world are being increasingly integrated with other data sources such as EHRs, we can believe that our models could be retrained to leverage newly linked databases, with increased discriminative performance. However, as it has been shown with EHRs^64^, one must always keep in mind that AHD reflect not only the health state of a patient but also the interactions they had with the healthcare system. Our temporal sliding window framework is robust to the bias of events in the administrative data reflecting past true health states (time of diagnosis is posterior to the time when symptoms started). Our model learns correlations between observed events and target adverse outcomes. Most of these correlations are not causal, and cannot be used to explain why a specific outcome has occurred. Inferring causal relationships would require a different conceptual and analytic framework, which is for future work^65^. Finally, as with other predictive models, external validation with recalibration and prospective validation as well as monitoring for distribution shifts over time would be important to conduct prior to widespread implementation and adoption.

In conclusion, we outline the development and validation of a machine learning model to predict adverse outcomes due to a range of diabetes complications three years ahead at the population level using routinely collected administrative data. We believe that after such models are externally and prospectively validated, public health officials will have a powerful tool for the ongoing risk assessment and cost-effective targeting of prevention efforts and resource allocation related to diabetes complications care at a population-scale.

## Methods

### Data source

This study was undertaken using publicly funded administrative health service records linked with population and other data holdings for Ontario, Canada. In Ontario, all residents are eligible for universal health coverage, so AHD covers virtually every Ontarian. Moreover, Ontario is Canada’s most populous province and among the most ethnically diverse populations in the world (Supplementary Table 4). In 2016, it had a population of 13.2 million (14.5 million in 2019), of which almost 30% were immigrants^66^.

The data were accessed at ICES, which is an independent, nonprofit research institute, whose legal status under Ontario’s health information privacy law allows it to collect and analyze healthcare and demographic data, without consent, for health system evaluation and improvement. We analyzed the data within the Health AI Data Analytics Platform (HAIDAP), a platform with high-performance computing resources required for advanced analytics.

The study used multiple diverse data sources including demographic information, census, physician claims, laboratory results, prescription medication history, hospital and ambulatory usage and others. These data sources were linked using the unique encoded identifiers from the Registered Persons Database (RPDB). The RPDB is a central population registry of all residents in Ontario who have ever received a health card number from the province’s universal single-payer healthcare system. This registry enables linkage across datasets, and contains basic demographic information, including sex, age, and geographical residence information that we used in our model.

Patients with diabetes were identified using the Ontario Diabetes Database (ODD), a validated registry of Ontario residents diagnosed with diabetes^42^. For each identified patient we extracted data on healthcare utilization and services accessed from the following sources: physician and emergency claims from the Ontario Health Insurance Plan (OHIP), hospitalization history from the Discharge Abstract Database (DAD), emergency services from the National Ambulatory Care Reporting System (NACRS) and prescription medication claims for individuals aged 65 years or above and those receiving social assistance. Diabetes-related laboratory test results were obtained from the Ontario Laboratory Information System (OLIS). The Ontario portion of the Immigration Refugees and Citizenship Canada (IRCC) permanent resident database was used to identify immigration status and country of birth. Neighbourhood-level measures of socioeconomic status, such as ethnicity marginalization score and material deprivation marginalization score shown in Table 1, were obtained using data from the 2001, 2006, and 2011 Canadian censuses (ON-Marg)^67^. Finally, patient deaths that occurred during the observation period were identified from the Office of the Registrar General-Deaths (ORG-D) database. A detailed description of all the data sources can be found in the Supplementary Table 1.

### Cohort and exclusion criteria

We used an eleven-year time period from Jan 1, 2006 to Dec 31, 2016 for this study. The Ontario Diabetes Database contained 1,645,089 patients that were flagged as being diagnosed with diabetes at some point in their life and alive on Jan 1, 2006. The algorithm used to identify these patients has demonstrated a sensitivity of 86% and a specificity of 97% compared to physician-assigned diagnoses identified in chart audits^68^.

We excluded patients that were not alive as of January 1, 2012 (*n* = 56,345), and immigrant patients who arrived in Canada later than the last test observation window (n = 21,108). This resulted in the final cohort of 1,567,636 patients, corresponding to more than 95% of the original cohort. Unlike previous studies that generally apply extensive selection criteria^69^, we only excluded forced conditions where the patient is either deceased or not in the system at the time of prediction.

### Study design

For each patient in the cohort we partitioned the 11-year time period into patient-time instances that represent a view of the patient at a specific point in time. Each instance was then assigned a 2-year observation window, 3-year buffer, and 3-month target window. We used nonoverlapping target windows so the first instance has an observation window [Jan 1, 2006–Dec 31, 2007], buffer [Jan 1, 2008–Dec 31, 2010], and target window [Jan 1, 2011–Mar 31, 2011]. Similarly, the last instance in our time period has an observation window [Oct 1, 2011–Sept 31, 2013], buffer [Oct 1, 2013–Sept 31, 2016], and target window [Oct 1, 2016–Dec 31, 2016]. Taking into account observation window and buffer time offsets, each patient can have up to 24 instances with nonoverlapping target windows in our 11-year time period. Following the exclusion criteria we removed all instances where the patient is not alive at the end of the target window (*n* = 1,611,222). We also excluded instances for immigrant patients where the landing date was after the start of the observation window (*n* = 259,911). Statistics on the resulting mean number of instances per year can be found in Supplementary Table 8. In addition to this setup, we experimented with the buffer sizes of one and five years. Performance results for these settings are shown in the Supplementary Figs. 1–4, 6, and 7, and the associated feature contributions are shown in Supplementary Figs. 8 and 9.

The health event data from the observation window is used to extract features that summarize a patient’s health history at that point in time. We found that the 2-year window was sufficient to obtain the necessary information. There is a sweet spot to find between having an observation window long enough to extract meaningful information, and generating enough instances to train the model with. Indeed, as the observation window grows wider, sliding it through our eleven years time period with three months gaps generates less and less instances, thus reducing the model input size and decreasing performance. Two years was found to be an ideal compromise in our early experiments, and was used thereafter.

The extracted features are then fed to the model that generates instance-level adverse outcome predictions. An instance is considered to have an adverse outcome if there is a corresponding event in its target window. This means that there is at least one hospitalization or ambulatory episode flagged with an ICD-10 code related to one of the diabetes complications during the target window. Adverse outcomes are used here since from AHD, we cannot necessarily ascertain when a complication first became apparent, but rather when an individual sought care for that complication. See Supplementary Table 5 for the list of ICD-10 codes used for adverse outcomes from each complication. Refer to Supplementary Tables 9–11 for statistics on the resulting adverse outcomes, including mean duration and incidence.

An overview of our approach is displayed in the Supplementary Methods, while we delve into details of our end-to-end pipeline in the diagram of Fig. 1. This justifies our choice of using a similar machine learning approach. The multi-instance approach simulates continuous population screening in a practical application. Specifically, we simulate a system where the entire cohort with diabetes is screened every 3 months, and most at-risk patients identified by the model are selected for further analysis and action. The main task is thus to accurately capture all instances that have an adverse outcome in the target prediction window. To achieve this the model must perform well across patients in the cohort and across time for each patient.

### Cohort partitioning

We partitioned the cohort into nonoverlapping sets of patients with 1,029,366 patients for model training then 272,864 and 265,406 patients for validation and testing, respectively. Patients in each set are selected at random. All model developments and parameter selections were performed on the training and validation sets, and we report the final model performance on the test set. To reduce the time bias we further partitioned the data in time. For patients in the training set we used instances that have target windows in [Jan 1, 2011–Dec 31, 2014]. Similarly, for validation and test sets we only kept instances with target windows in [Jan 1, 2015–Dec 31, 2015] and [Jan 1, 2016–Dec 31, 2016], respectively. The detailed statistics for each set are summarized in Table 1. Partitioning in time ensures that there is no overlap between the sets so all testing is performed both out-of-sample and out-of-time. This provides a more accurate estimate of performance since in practice, the model would be applied to patients who are newly diagnosed with diabetes (out-of-sample), and all predictions would be done forward in time compared to the training data (out-of-time).

### Feature extraction

The main features that we examined were derived from demographics (not changing over time), geographical information, chronic conditions and healthcare utilization history. Stationary features included sex, birth year, immigrant status, and country of origin. Geographical information comprised residence statistics and measures of area-level socioeconomic status from recent census surveys at the level of the first three digits of the postal code. healthcare utilization included information on physician/specialist visits, hospitalization and ambulatory usage and prescription history as seen in Fig. 3. It comprised emergency department visits and laboratory results during the observation window.

We did not perform any preprocessing of continuous variables, except for laboratory results. Laboratory results can be reported in different units, such as mg/L and g/L, and we standardized the unit before doing feature extraction. One-hot encoding was used for all categorical variables, and we discarded categories that appeared with a frequency of less than 1%. Removing infrequent categories significantly reduced the feature size and improved model generalization.

As reported in previous studies, we also found that events in the observation window occur in highly irregular patterns^70,71^. Patients would typically have clusters of activity (multiple doctor/ER visits, laboratory tests, etc.) followed by quiet periods with few events. To summarize these patterns we performed various aggregations over different time intervals within the two year observation window. For time aggregation we counted events in the last month, quarter, 6 months, year, etc. For event aggregation we combined events of the same type such as doctor visits by physician specialty and prescription medication by drug type. This double aggregation resulted in features such as “number of ophthalmologist visits in the last month” and “total quantity of drug X prescribed in the last year”. We found such features to be highly informative for adverse outcomes prediction, and many of them appear in the top features as seen in Fig. 3. During feature selection we adopted a greedy approach, and computed multiple combinations of time and event aggregation. These features were then incrementally added into the model and retained only if the validation set performance improved. Note that throughout this process, to prevent any model bias, the test set remained untouched and was only used for the test performance computation of the final model.

In addition to event aggregation, we included other features that summarize a patient’s recent medical history. To estimate the recurrence frequency we computed time between consecutive events, as well as time since the most recent event. The goal was to estimate whether certain events are becoming more frequent or occur with a specific time pattern. We particularly focused on the past diabetes and diabetes-related complications as these are generally indicative of future complications. Moreover, we compared each patient’s event history with histories from patients in the same sex, age, and immigration status groups. Within-group comparisons can identify “outlier” patients whose progression of condition trajectory deviates significantly from other patients^72,73^. All feature selection here was performed in a similarly greedy fashion by incrementally adding subsets of features to the model. After multiple rounds of feature selection we obtained a set of ~700 features that maximized the validation AUC, and used this set for all further experiments. Supplementary Table 2 provides additional details on feature engineering while Table 3 provides a guide for reading the feature names.

### Model development

We trained the Gradient Boosting Decision Trees (GBDT) model implemented in Python in the XGBoost open source library^74,75^. GBDT was chosen due to its ability to handle different feature types (categorical, ordinal, numerical, missing values, etc.) as well as good support for explainability. Besides, XGBoost coupled with extensive feature engineering has consistently shown extremely competitive performance on tabular data. It was used in numerous winning solutions to Kaggle competitions^76^ and ACM Recommender Systems Challenges^77,78^, and was also proven successful on longitudinal healthcare data^79^. We also experimented with leading deep learning models such as recurrent neural networks including GRU-D^80^, multilayer perceptron and Transformers with self-attention^80,81^. However, XGBoost consistently outperformed these models by a relatively large margin. This aligns with previous findings on similar heterogeneous tabular datasets^82,83^. Details on the comparison with logistic regression can be found in the Supplementary Methods and the logistic regression model’s performance in Supplementary Table 7.

To handle the multi-class problem of predicting adverse outcomes for multiple complications, we adopted the Cross-Class Relevance Learning (CCRL) method, where class index is appended to the input features and the task is transformed into binary classification^84^. This significantly accelerated training since otherwise we require to optimize a separate XGBoost model for each class, i.e., one for each complication. Our model outputs five risk scores (one per complication) for each instance that is fed to it.

To find good settings of hyperparameters we ran grid search by first specifying ranges for each hyperparameter, and then exhaustively evaluating on points selected from those ranges. After grid search we selected the following settings: a tree depth of 10, learning rate of 0.05, minimum child weight of 50, alpha = 0.3, gamma = 0.1, lambda = 0.0, column sample by tree of 0.6 and column sample by level of 0.6 (relevant XGBoost parameter documentation can be found at: https://xgboost.readthedocs.io/en/latest/parameter). Since incidence rates for adverse outcomes are typically lower than 1%, we undersampled negative instances by a factor of up to 10× to balance the training data^85,86^. After training, the output probabilities from the model were recalibrated using the approach proposed by Pozzolo, et al.^87^.

### Model evaluation

Given the large-scale size of our final cohort (millions of patients, tens of millions of instances), we do not use k-fold cross-validation and stick to a single, fixed validation set, as is common practice with very large datasets^43,44^. Our validation set is large enough to capture the whole population distribution. We evaluated the test performance of our model on a distinct held out test set using the Area Under the Receiver Operating Curve (AUC) metric. AUC is robust to significant label imbalance^88^, and is commonly used for such prediction tasks^89^. Calibration of the model was assessed by plotting calibration curves of the observed versus the predicted probabilities across 20 evenly partitioned bins. The calibration curves and AUC results for each complication are shown in Fig. 2. For practical application, we are particularly interested in the most at-risk patients predicted by the model. As we discussed earlier, such patients can be further analysed for possible preventative measures and resource management. To evaluate performance for the top predicted patients, we also computed the precision (positive predictive value) and recall (sensitivity) shown in the Supplementary Figs. 1 and 2.

We used the Shapley values to find top features that contribute the most to model prediction^46^. To estimate the contribution for each feature we averaged absolute Shapley values over a sample of 100,000 instances selected at random from the test cohort. Different samples were used for each complication to avoid biasing the estimates.

### Costing methodology

To evaluate whether the model can capture a significant portion of the cost associated with treating adverse outcomes^90^, we computed annual cost for most at-risk patients predicted by the model. Diabetes and its related complications place a significant cost burden on the healthcare system. Continuous population screening and early detection can lead to significant cost savings through preventative measures and resource planning. However, this would only be possible if the model can accurately predict the costly outcomes, meaning that it can make higher predictions on instances with costly adverse outcomes due to diabetes complications than on negative instances. The cost is computed by first applying the costing algorithm^49^ to estimate total annual healthcare expenditure by category (hospitalizations, prescriptions, etc.) for each patient. The costing algorithm follows a bottom-up approach for ambulatory care to get person-level healthcare expenditure per year and per category of healthcare utilization by mapping the utilization data with cost information. For inpatient hospitalizations, emergency department visits and same day surgery costs, the algorithm estimates costs based on average provincial costs for these procedures weighted by the resource intensity in a given care setting. Utilization data is directly available through the administrative databases leveraged in this study. Cost information is estimated in the algorithm based on amounts billed to the Ministry of Health and Long Term Care (MOHLTC). We used this costing algorithm off-the-shelf (without any tweaking) on our cohort. This algorithm has been previously validated and is further described elsewhere^47,91^. From the category estimates, we then isolated the portion of the cost attributed to adverse outcomes by isolating cost from the relevant hospitalizations and ambulatory usage. Finally, we sorted all patients according to the model predicted probability of adverse outcome, and computed cumulative cost for each percentages of this sorted list. Cost in one percentile is just the sum of costs of all patients in this percentile. Results for this analysis are shown in Fig. 4.

### Ethics

This study obtained ethics approval from the Research Ethics Board at the University of Toronto (Protocol # 37650).

### Reporting summary

Further information on experimental design is available in the Nature Research Reporting Summary linked to this paper.

## Supplementary information

Supplementary File

Reporting Summary

## Data Availability

The dataset for this study is held securely in coded from at ICES. While data sharing agreements prohibit ICES from making the dataset publicly available, access may be granted to those who meet prespecified criteria for confidential access, available at www.ices.on.ca/DAS. The full dataset creation plan is available from the authors upon request.

## Code availability

The data for this study was prepared with custom code from ICES using the SAS Enterprise v6.1 software. This data were later analyzed with custom code from Layer 6 AI in the Java 8 and Python 3.6 programming languages. The analytic code is available from the authors upon request, understanding that the computer programs may rely upon coding templates or macros that are unique to ICES and this data and thus may require modification.
