# Supplementary material for: Predicting adverse outcomes due to diabetes complications with machine learning using administrative health data
Source: NPJ Digit Med. 2021 Feb 12;4:24. doi: 10.1038/s41746-021-00394-8 (PMC7881135; doi:10.1038/s41746-021-00394-8)
Supplement: Supplementary file 1 — Supplementary File [file 41746_2021_394_MOESM1_ESM.pdf]

Predicting Adverse Outcomes Due to Diabetes Complications with Machine Learning Using Administrative Health Data

Mathieu Ravaut[1,2], Hamed Sadeghi[1], Kin Kwan Leung[1] , Maksims Volkovs[1],
Kathy Kornas[3], Vinyas Harish[3,4] , Tristan Watson[3,5], Gary F. Lewis[6,7],
Alanna Weisman[8,9], Tomi Poutanen[1], Laura Rosella[3,5,10-12] *

[1] Layer 6 AI, Toronto, ON, Canada
[2] Department of Computer Science, University of Toronto, Toronto, ON, Canada
[3] Dalla Lana School of Public Health, University of Toronto, Toronto, ON, Canada
[4] MD/PhD Program, Temerty Faculty of Medicine, University of Toronto, Toronto, ON, Canada
[5] ICES, Toronto, ON, Canada
[6] Department of Medicine, Temerty Faculty of Medicine, University of Toronto, Toronto, ON, Canada
[7] Department of Physiology, Temerty Faculty of Medicine, University of Toronto, Toronto, ON, Canada
[8] Lunenfeld-Tanenbaum Research Institute, Mt. Sinai Hospital, Toronto, ON, Canada
[9] Division of Endocrinology and Metabolism, Temerty Faculty of Medicine, University of Toronto, Toronto, ON, Canada
[10] Vector Institute, Toronto, ON, Canada
[11] Institute for Better Health, Trillium Health Partners, Mississauga, ON, Canada
[12] Department of Laboratory Medicine & Pathology, Temerty Faculty of Medicine, University of Toronto, Toronto, ON, Canada

* Indicates corresponding author (laura.rosella@utoronto.ca)

Supplementary Figures

Figure 1: Precision Curves. Precision curves for all buffer sizes.

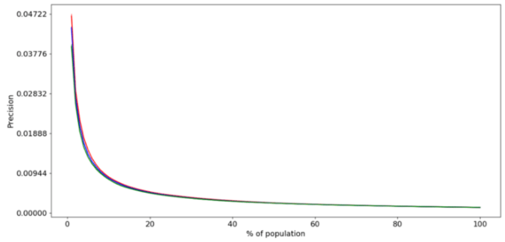

(a) Hyper/hypo - glycemia

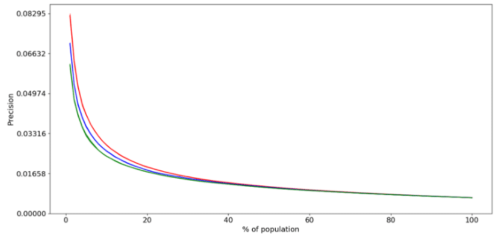

(b) Tissue infection

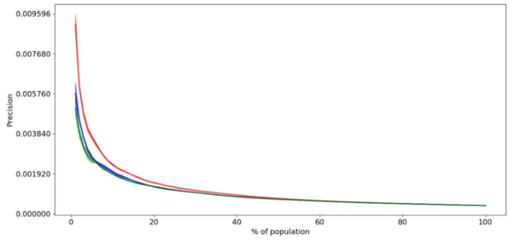

(c) Retinopathy

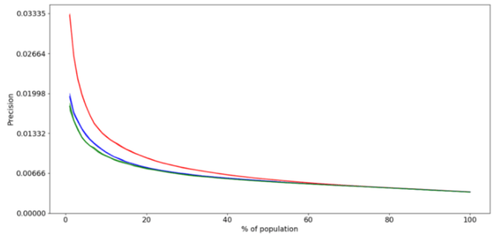

(d) Cardio-vascular events

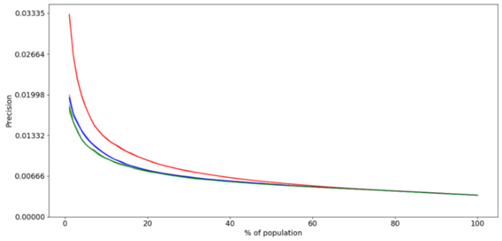

(e) Amputation

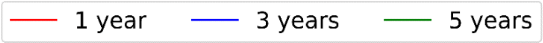

Figure 2: Recall Curves. Recall curves for all buffer sizes.

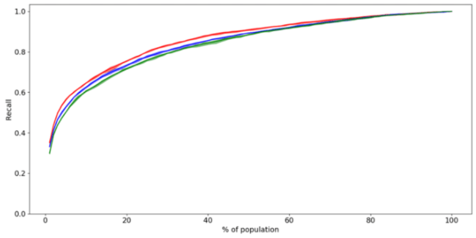

(a) Hyper/hypo - glycemia

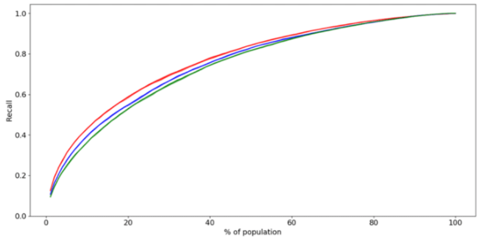

(b) Tissue infection

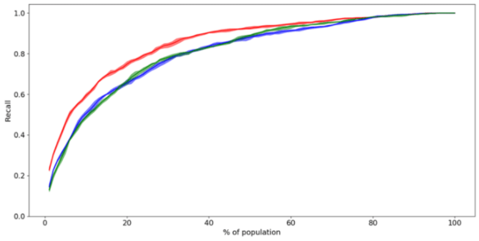

(c) Retinopathy

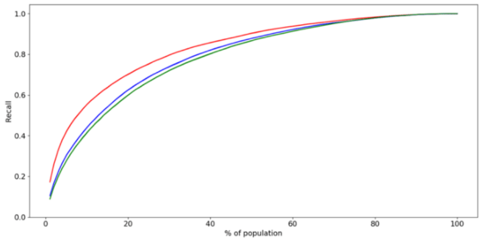

(b) Cardio-vascular events

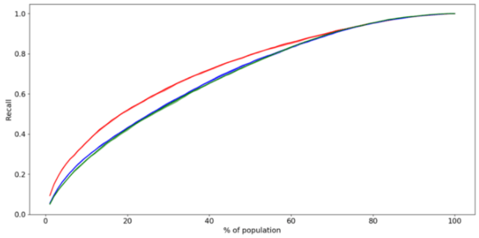

(e) Amputation

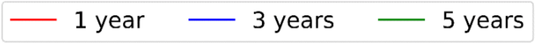

Figure 3: Specificity Curves. Specificity curves for all buffer sizes.

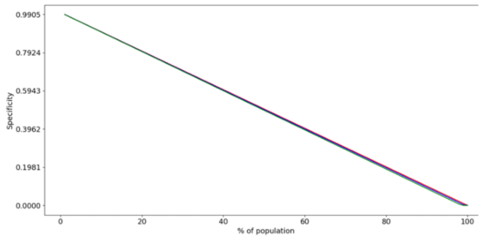

(b) Hyper/hypo - glycemia

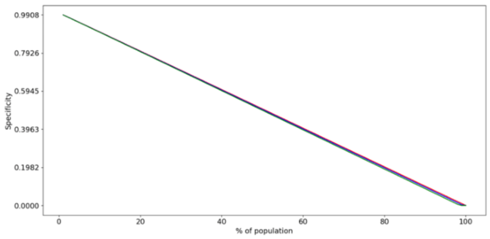

(a) Tissue infection

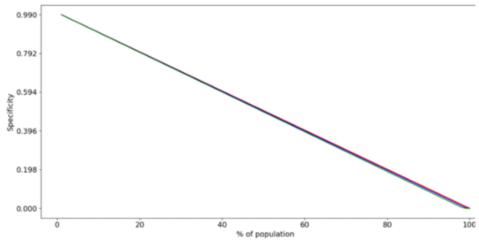

(c) Retinopathy

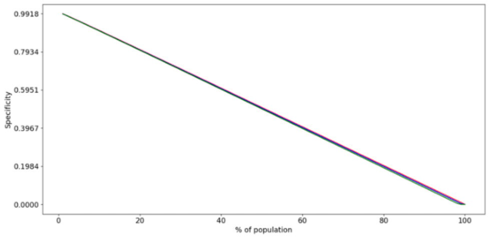

(d) Cardio-vascular events

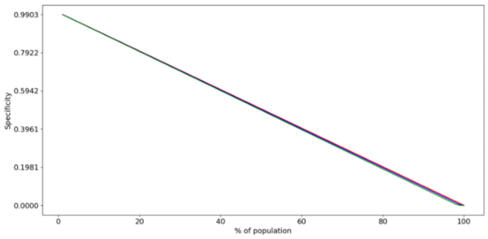

(e) Amputation

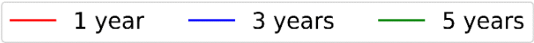

Figure 4: Negative Predictive Values Curves. Negative predictive values curves for all buffer sizes.

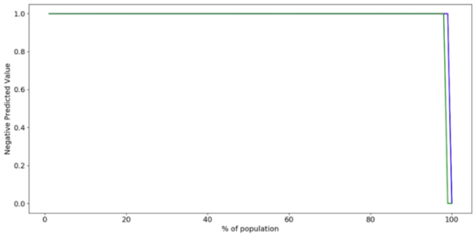

(a) Hyper/hypo - glycemia

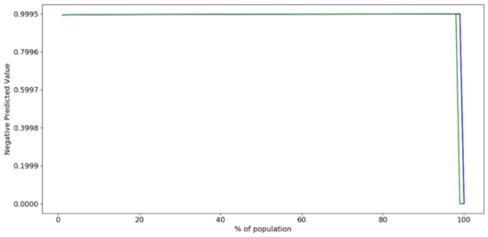

(b) Tissue infection

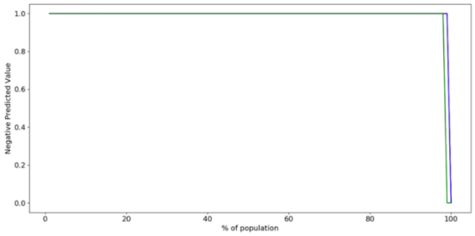

(b) Retinopathy

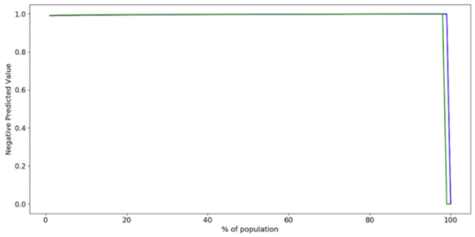

(d) Cardio-vascular events

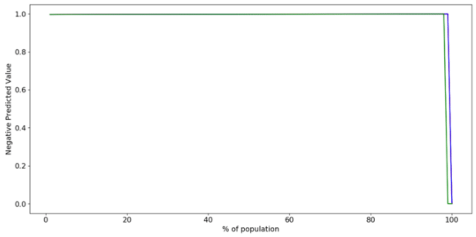

(e) Amputation

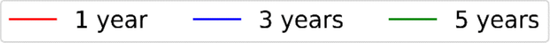

Figure 5: Model Performance over Different Population Subgroups. Model performance is computed across sex, age immigration status and event density subgroups. The red vertical axes correspond to the percentage of test instances in each subgroup. Histograms on the right show fraction and count of instances in each subgroup that have adverse outcomes. Due to very low incidence rates in some subgroups, complications that have fewer than 30 adverse outcomes in any subgroup are excluded from the AUC calculation. The "Age < 20" subgroup is also excluded from the analysis due to a too small incidence rate.

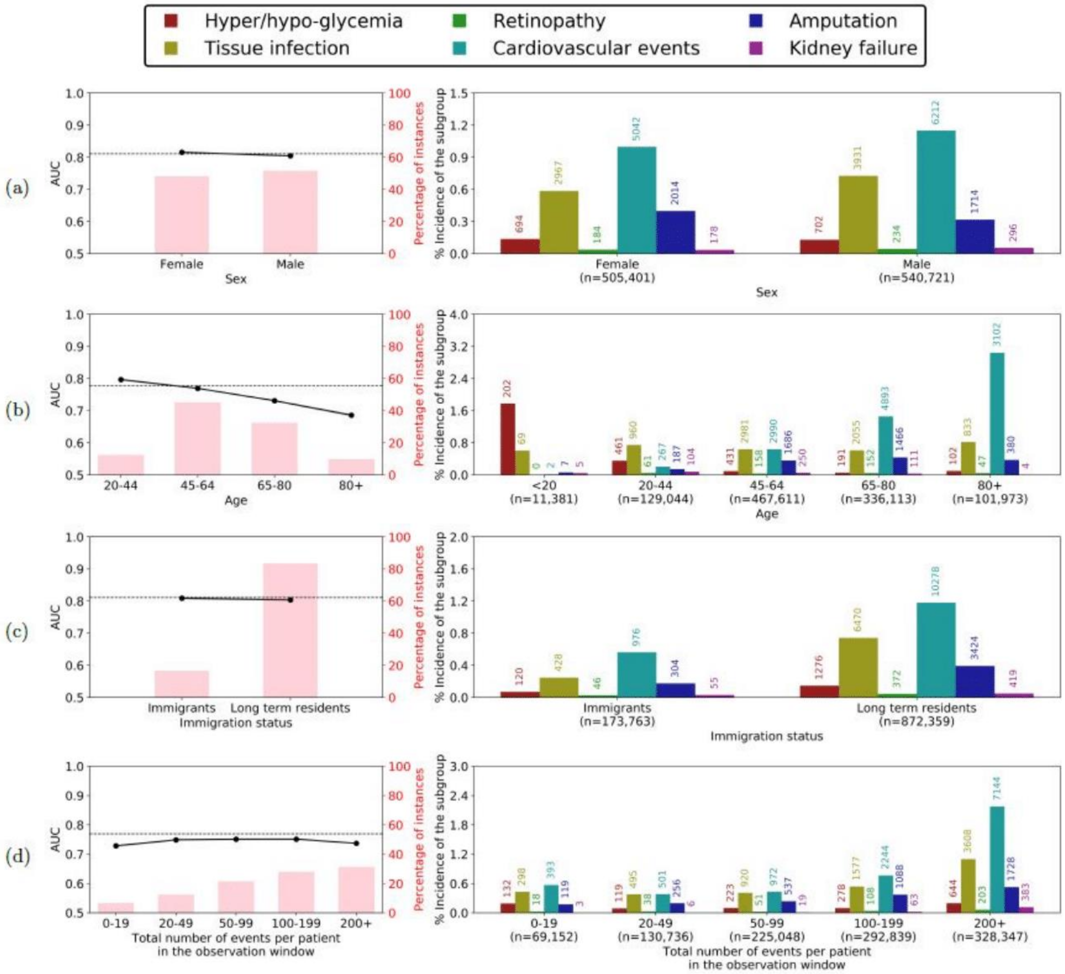

Figure 6: Model Performance for a Buffer of One Year.

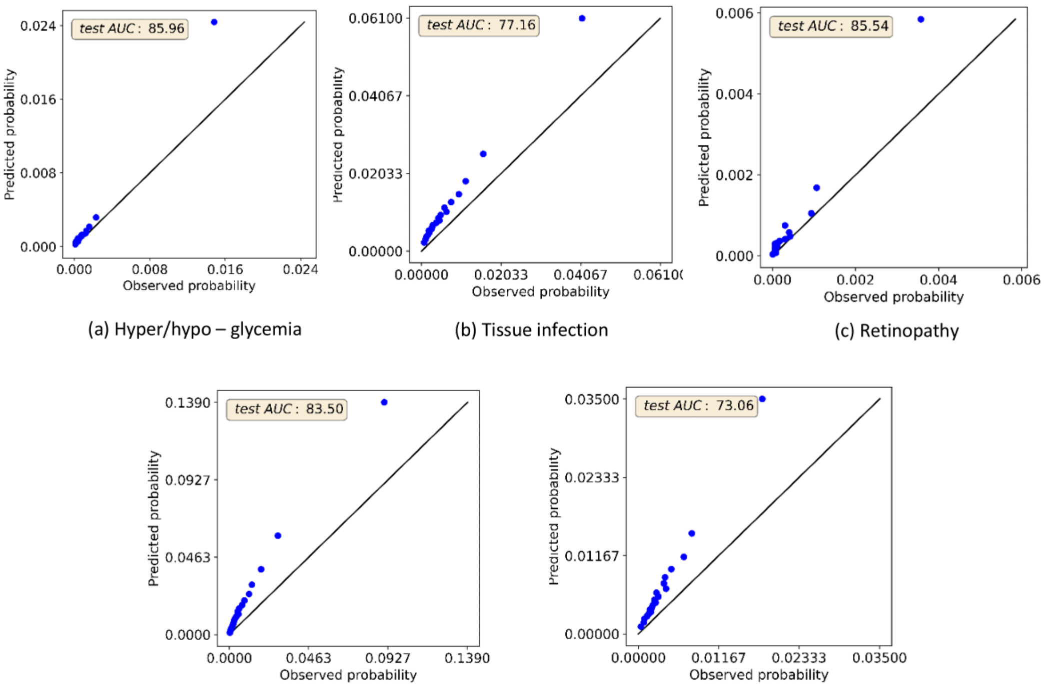

(a) Hyper/hypo – glycemia

(b) Tissue infection

(c) Retinopathy

(d) Cardiovascular events

(e) Amputation

Figure 7: Model Performance for a Buffer of Five Years.

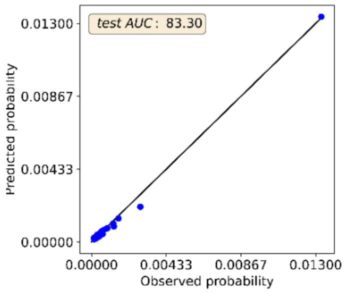

(a) Hyper/hypo – glycemia

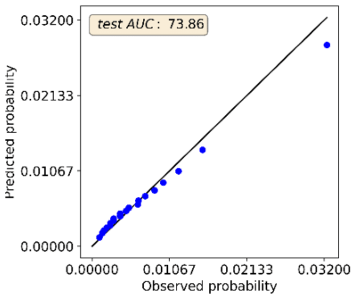

(b) Tissue infection

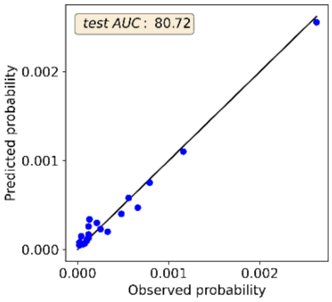

(c) Retinopathy

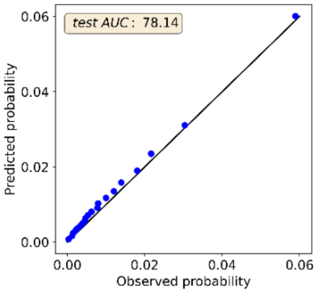

(d) Cardiovascular events

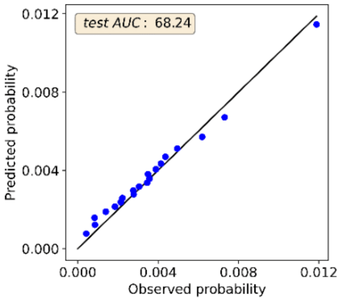

(e) Amputation

Figure 8: Feature Contribution for a Buffer of One Year. Top eight feature contribution on the test set for each complication.

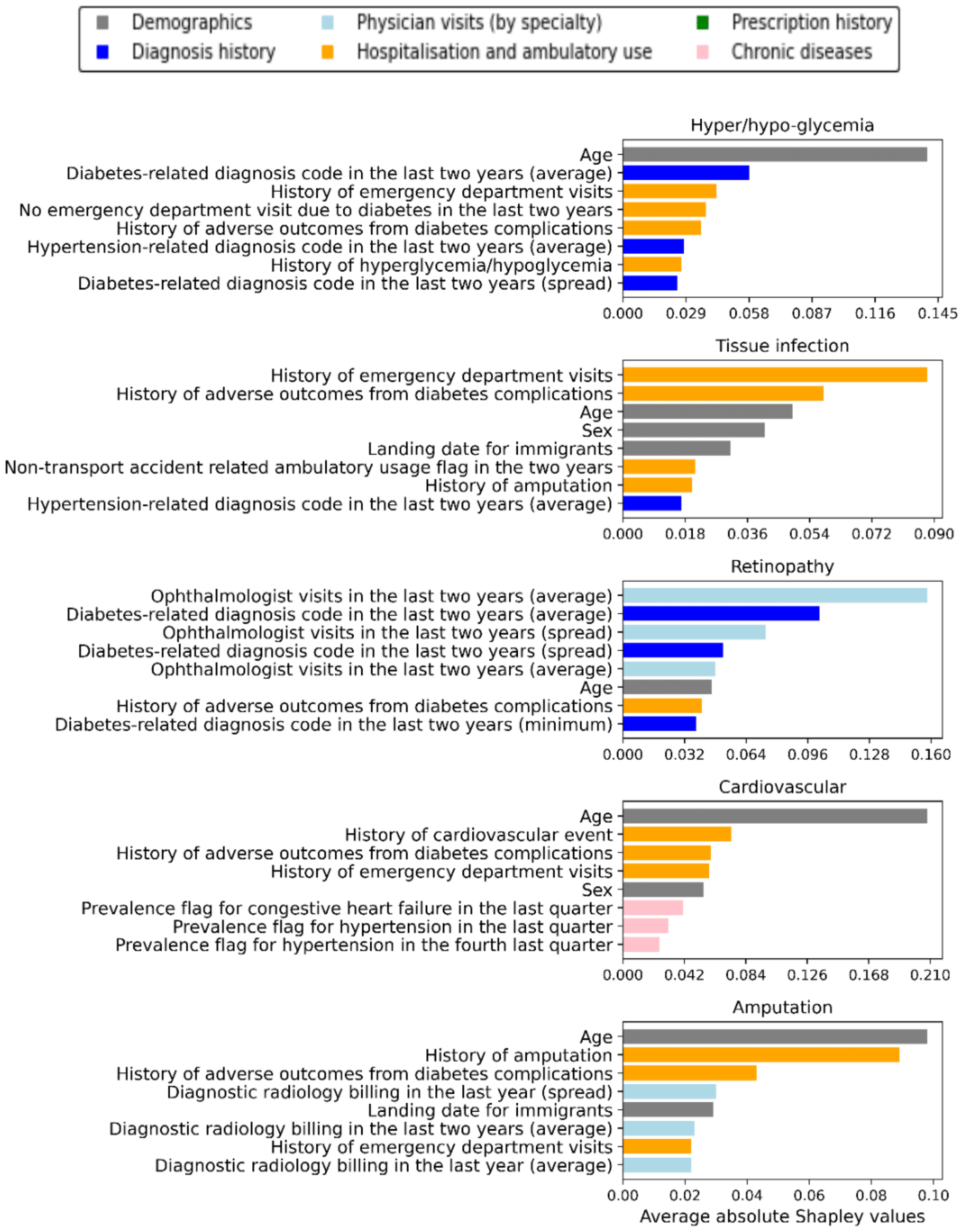

Figure 9: Feature Contribution for a Buffer of Five Years. Top eight feature contribution on the test set for each complication.

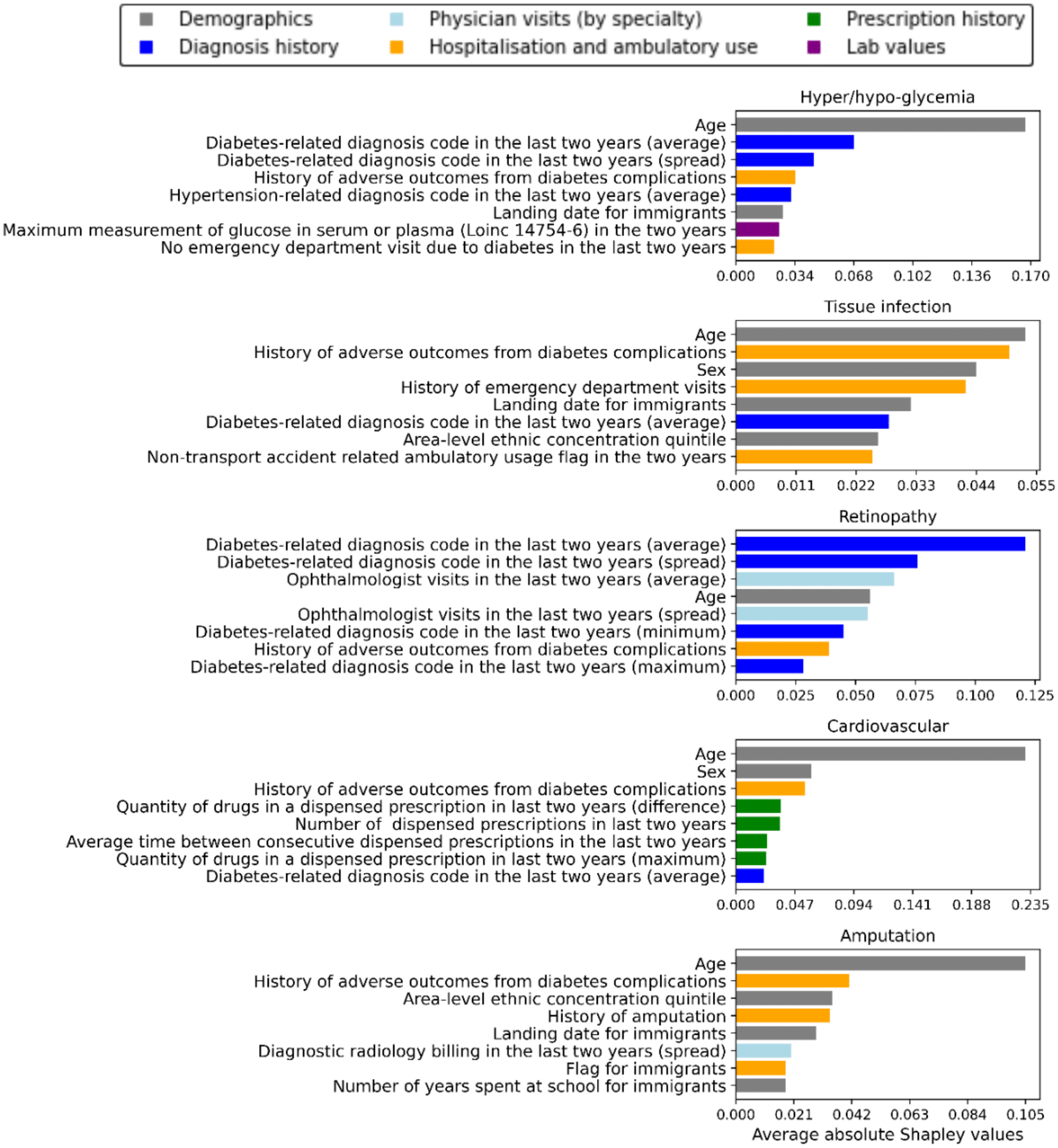

Supplementary Tables

Table 1: Dataset Description. Here, we provide more details regarding the used datasets. ICES hosts approximately 100 different datasets, and we used 19 of them capturing possibly all aspects related to diabetes

Dataset	Description
**Registered Persons Database (RPDB)**	Provides basic information about anyone who have ever received an Ontario health card number. Key data variables include date of birth, sex, geographical information, and time periods for which an individual was eligible for coverage under the Ontario Health Insurance Plan (OHIP). All health card numbers are encoded before being linked to other databases at ICES.
**IRCC Permanent Residents Database (CIC)**	Contains records for over three million individuals at the time of landing in Ontario from January 1985 to May 2017, and is linked to the RPDB with a 86.4% overall linkage rate [a]. Data include permanent residents' demographic information such as country of birth and landing date.
**Ontario Census Area Profile (CENSUS)**	Self-reported information collected during population census in 2001 and 2006.
**Ontario Marginalization Index (ONMARG)**	Socio-economic neighborhood information collected in 2001 and 2006 census.
**Postal Code Conversion File (PCCF)**	Geographical information such as latitude or longitude.
**Local Health Integration Net-work (LHIN)**	Describe which LHIN does the patient refer to. Ontario is partitioned into 14 different LHINs.
**REF**	Further stationary data such as sex.
**Ontario Health Insurance Plan (OHIP)**	The OHIP claims database contains information on inpatient and outpatient services provided to Ontario residents eligible for the province's publicly funded health insurance system by fee-for-service health care practitioners (primarily physicians) and shadow billings for those paid through non-fee-for-service payment plans. The main data elements include patient and physician identifiers (encrypted), code for service provided, date of service, associated diagnosis, and fee paid. We also extracted OHIP emergency claims data using OHIP Emergency Services (ERCLAIM) dataset, which uses a macro to extract emergency claims data from OHIP claims (one record per emergency service).
**Ontario Drug Benefits Claims (ODB)**	The ODB database contains prescription medication claims for those covered under the provincial drug program, mainly: those aged 65 year and older, nursing home residents, patients receiving services under the Ontario Home Care program, those receiving social assistance, and residents eligible for specialized drug programs. Main data elements include drug identifier, quantity, number of days supplied, date dispensed, cost, and patient, pharmacy and physician identifiers.
**Discharge Abstract Database (DAD)**	The DAD is compiled by the Canadian Institute for Health Information and contains administrative, clinical (diagnoses and procedures/interventions) and demographic information for all admissions to acute care hospitals, rehabilitation, chronic, and day surgery institutions in Ontario. At ICES, consecutive DAD records are linked together to form episodes of care among the hospitals to which patients have been transferred after their initial admission.
**National Ambulatory C are Reporting System (NACRS)**	The NACRS is compiled by the Canadian Institute for Health Information and contains administrative, clinical (diagnoses and procedures), demographic, and administrative information for all patient visits made to hospital- and community-based ambulatory care centers (emergency departments, day surgery units, haemodialysis units, and cancer care clinics). At ICES, NACRS records are linked with other data sources (DAD, etc.) to identify transitions to other care settings, such as inpatient acute care or psychiatric care.
**Ontario Laboratory Inf ormation System (OLIS)**	A system that connects hospitals, community laboratories, public health laboratories and practitioners to facilitate the secure electronic exchange of laboratory test orders and results. This database provides results for routine laboratory tests, including HbA1c, lipids, serum, creatine, and albumin/creatine ratio from 2006 onwards.
**OHIP's Emergency Claims Database (ERCLAIM)**	More specific details for emergency claims.
**Ontario Diabetes Database (ODD)**	As stated, in our study, we identified patients living with diabetes from a validated ICES derived registry of all Ontarians identified as having diabetes (prevalent cases) since 1991, which demonstrated sensitivity of 86% and a specificity of 97%. An individual is flagged with diabetes (and included in ODD) if one of the following conditions is met: (1) Two OHIP (physician) claims with diabetic diagnostic code within a two-year period; (2) One hospitalization (in DAD) with diabetic diagnostic code; or (3) A single OHIP claim with a fee code for diabetes management, insulin therapy support, diabetes management assessment. Such code belongs to the following list: Q040, K029, K030, K045 and K046 [b]. The diabetes diagnostic codes are given by ICD-9 250.xx or ICD-10 E10-E14.
**ASTHMA, CHF, HYPER, OCCC, OMID, ORAD**	Derived cohorts for the six respective chronic diseases: asthma, congestive heart failure, hypertension, Crohn's disease, myocardial infarction and rheumatoid arthritis. These datasets contain yearly binary flags for both prevalence and incidence of the associated disease for the patient.

[a] Maria Chiu et. Al. Describing the linkages of the Immigration, Refugees and Citizenship Canada permanent resident data and vital statistics death registry to Ontario's administrative health database. BMC medical informatics and decision making, 16(1):135, 2016.
[b] We used the 2016 version of the ODD.

Table 2: Feature Engineering. We detail all the features that we manually designed, for each category of variables.

**Geographical**	
Distance to LHIN	Geodesic distance to the closest LHIN
**Observation**	
HbA1c	For diabetes related features, we isolated the HbA1c laboratory results using the LOINC(codes), because several LOINCs correspond to HbA1c: 4548-4, 71875-9, 59261-8, 17855-8, 17856-6 and 41995-2.
Length of stay	We created the length-of-stay for the DAD observations by subtracting discharge time and admittance time.
**ICD codes**	
ICD chapters	ICD chapter numbers for several diagnosis code features in the ICD format: DAD-dx10code, NACRS-dx10code (ICD-10) and OHIP-dxcode, ER-dxcode (ICD-9).
Diabetes code	Among the ICD codes in the set, is there one linked to diabetes?
**Observations frequency**	
Counts	Number of observations per dataset per quarter.
Cumulative counts	Cumulative number of observations (since the last two years) per dataset.
Time since	Time elapsed (in seconds) since last observation per dataset within two years.
Average frequency	Average time between consecutive observations from this dataset within two years.
Standard deviation of frequency	Standard deviation of times between consecutive observations from the dataset during the last two years.
**Complications history**	
Any complications	Presence of any complications, at each quarter, over the last two years.
Complications	Complications at each quarter within two years.
Cumulative labels	Total number of any complications until now.
Cumulative backwards	Cumulative number of complications from the quarter to now, per complication, within two years.
Time since last complication	Time elapsed (in quarters) since any last complication, within two years.
Time since	Time elapsed (in quarters) since the last complication, per complication, within two years.
**Time**	
Quarter	Quarter of the year, for the last quarter of the observation window
**Statistics at the population level**	
Distance to sex average	Difference between patient number of observations and the average number of observations over the patients with the same sex (2 groups), per dataset.
Distance to age average	Difference between patient number of observations and the average number of observations over the patients within the same age group (same groups as in Results), per dataset.
Distance to immigration group average	Difference between the patient's number of observations and the average number of observations over the patients within the same immigration group (2 groups), per dataset.

Table 3: Feature Name Guidelines. We describe the guidelines reading the feature names listed in the feature contribution tables.

**Features**	
Number of years spent at school for immigrants	Values are assigned missing for long term residents.
Landing date for immigrants	Values are assigned missing for long term residents
OHIP diabetes-related code	ICD-9 code of 250.xx in the OHIP dataset.
OHIP hypertension-related code	ICD-9 code of 401.xx in the OHIP dataset.
Transport accident related	ICD-10 code starts with V in the NACRS dataset.
History of diabetes complications	Any complications identified in ICD-10 codes listed in Supplementary Material Table V.
**Aggregators**	
(Average)	The average value in the category over the given period.
(Spread)	The standard deviation of the values in the category over the given period.
(Minimum)	The minimum value in the category over the given period.
(Maximum)	The maximum value in the category over the given period.
(Difference)	The difference between the maximum and the minimum values in the category over the given period
(Amplitude)	The difference between the last and the first values in the category over the given period

Table 4: Top 15 Most Frequent Countries of Birth. We only display the top 15 as the full list of countries exceeds 60. These countries together cover more than 80% of all immigrants.

Country of birth	Number of patients	Fraction of immigrants (%)
India	54,145	22.1
Pakistan	24,434	10.0
Bangladesh	23,975	9.8
Afghanistan	17,934	7.3
Jamaica	16,472	6.7
Sri Lanka	11,270	4.6
Philippines	11,048	4.5
Hong Kong	6,684	2.7
Somalia	6,573	2.7
Poland	5,371	2.2
Iran	5,278	2.2
China	5,158	2.1
Germany	4,573	1.9
Portugal	4,382	1.8
South Korea	3,771	1.5

Table 5: Outcomes Definition. The list of ICD10/CCI codes from DAD and NACRS used to determine adverse outcomes for each diabetes complication.

Complication	ICD10/CCI codes
Hyper/Hypo - glycemia	E10.0, E10.1, E11.0, E11.1, E13.0, E13.1, E14.0, E14.1, E16.0, E16.1, E16.2, E08.65, E08.01, E08.641, E13.641, E11.65, E10.65, E11.641, E10.641
Tissue infection	L00, L01, L02, L03, L04, L05, L08, M72.5, M72.6, A48.0, E10.51, E11.51, E13.51, E14.51, R02, E10.61, E11.61, E13.61, E14.61, E10.70, E11.71, E13.71, E14.71, E08.620, E08.621, E08.622, E08.628, E09.620, E09.622, E09.628
Retinopathy	E10.31, E10.32, E10.33, E10.34, E10.35, E10.36, E11.31, E11.32, E11.33, E11.34, E11.35, E11.36, E13.31, E13.32, E13.33, E13.34, E13.35, E13.36, E08.311, E08.319, E08.36, E08.39
Cardiovascular events	I21, I22, I61, I63, I64, I50, I20, G450, G45.3, G45.8, G45.9
Amputation	1VC, 1VG, IVQ, 1WA, 1WE, 1WI, 1WJ, 1WK,1WL, 1WM, 1WN

Table 6: Electronic Medical Records (EMR) and Administrative Health Data (AHD). We compare the content of our input data with previous studies using Electronic Medical Records (EMR). Note that these studies do not tackle prediction of adverse outcomes from diabetes complications but prediction of diabetes onset. Typically, AHD lacks the presence or coverage over all patients for key variables, especially among laboratory values.

Study	Genetic information	Family history	Lifestyle, health surveys	Demographics			Comorbidities	Drug history	Diagnosis history	Laboratory values			
				Ethnicity	Socio-economic	Geography	Hypertension			BMI or weight	HbA1c	Glucose	Triglycerides
**EMR**													
Cahn (2020)	No	No	No	No	No	No	No	Yes	No	Yes	Yes	Yes	Yes
Choi (2019)	No	Yes	Yes	Yes	Yes	No	Yes	Yes	No	Yes	Yes	Yes	No
**AHD**													
Razavian (2015)	No	No	No	No	No	No	Yes	Yes	Yes	Yes, partially	Yes, partially	Yes, partially	Yes, partially
Ours	No	No	No	No, only marginalization	Yes	Yes	Yes	Yes, partially	Yes	No	Yes, partially	Yes, partially	Yes, partially

Table 7: Logistic Regression Discrimination (AUC) Test Results. We test logistic regression models on the same test set and with the same input features as the XGBoost model.

Complication	1 year buffer	3 years buffer	5 years buffer
Hyper/hypo-glycemia	82.6 (82.2-82.8)	81.3 (81.0-81.5)	80.2 (80.0-80.6)
Tissue infection	76.7 (76.6-76.8)	74.1 (74.1-74.2)	72.4 (72.3-72.5)
Retinopathy	82.6 (82.5-82.8)	79.1 (78.7-79.4)	78.0 (77.5-78.4)
Cardiovascular events	72.5(72.4-72.8)	78.6 (78.6-78.7)	77.3 (77.3-77.4)
Amputation	79.5 (79.3-79.6)	76.1 (75.9-76.2)	65.9 (65.6-66.2)
**Mean**	**79.49 (79.3-79.6)**	**76.08 (75.9-76.2)**	**74.76 (74.5-75.0)**

Table 8: Distribution of the mean number of instances per patient per year. We show the mean number of instances (as defined in Methods) used per patient within one year for each of the training, and validation sets, as well as for the whole population and splits of the population on several attributes: sex, age group and immigration status.

	Training set	Validation set	Test set
Whole population	3.96	3.95	3.94
**Sex**			
Male	3.96	3.95	3.94
Female	3.95	3.95	3.95
**Age group**			
< 20	3.98	3.98	3.96
20 – 44	3.97	3.97	3.95
45 – 64	3.98	3.97	3.96
65 - 79	3.95	3.94	3.94
80+	3.84	3.83	3.82
**Immigration status**			
Immigrant	3.93	3.93	3.93
Long-term resident	3.96	3.95	3.94
**Material deprivation marginalization score**			
1st quintile	3.96	3.95	3.95
2nd quintile	3.96	3.95	3.95
3rd quintile	3.96	3.95	3.94
4th quintile	3.95	3.95	3.94
5th quintile	3.95	3.95	3.94
**Ethnicity marginalization score**			
1st quintile	3.95	3.95	3.94
2nd quintile	3.96	3.95	3.94
3rd quintile	3.96	3.95	3.94
4th quintile	3.96	3.95	3.95
5th quintile	3.96	3.95	3.94

The mean number of instances is lower in the last age group because patients are more likely to die. The mean number of instances is also slightly lower for immigrants because some of them land in Canada after the beginning of the period and thus have less information available on them.

Table 9: Mean duration of adverse outcomes. When a patient has an adverse outcome from a given complication, the whole quarter during the which it happens is flagged as a positive instance. It is possible that the quarter immediately before or immediately after also have adverse outcomes. Here we show the mean number of consecutive quarters for a given adverse outcome episode, across all training validation and test sets. As seen mean durations are close to 1 since there is typically just a single quarter during the which an adverse outcome happens.

**Complication**	**Training set**	**Validation set**	**Test set**
Hyper/hypo-glycemia	1.37	1.15	1.17
Tissue infection	1.37	1.14	1.14
Retinopathy	1.22	1.12	1.08
Cardiovascular events	1.52	1.21	1.19
Amputation	1.23	1.06	1.07

Table 10: Fraction of positives before the last test instance. We show the average incidence throughout the test set of adverse outcomes in two setups: (*) at any time before the last test instance (which target window is the last quarter of 2016), and (**) in the quarter immediately before the last instance, which is the third quarter of 2016. In the first setup, we look back to our earliest available data, of January 1st 2006. We conclude that in the immense majority of cases, a patient does not have immediate prior adverse outcomes due to diabetes complications, and in the majority of cases, does not have at all prior history of adverse outcomes.

Complication	Positives any time before the last instance (%) *	Positives in the in the quarter before the last instance (%) **
Hyper/Hypo-glycemia	5.49%	0.19%
Tissue infection	20.01%	0.82%
Retinopathy	1.34%	0.03%
Cardiovascular events	33.19%	1.22%
Amputation	13.29%	0.33%

Table 11: Quarterly incidence rate (in %) in the target window for adverse outcomes from each complication for the training, validation and test sets. Complications (columns) are denoted by letters: A for hyper/hypo-glycemia, B for tissue infection, C for retinopathy, D for cardiovascular events and E for amputation. Rows represent quarters, where four quarters sum up to one year (separated by dashed lines).

	Training					Validation					Test				
	A	B	C	D	E	A	B	C	D	E	A	B	C	D	E
**Q1**	0.096	0.447	0.035	0.886	0.325										
**Q2**	0.089	0.524	0.034	0.934	0.340										
**Q3**	0.094	0.617	0.028	0.886	0.294										
**Q4**	0.104	0.543	0.039	0.994	0.361										
**Q5**	0.097	0.501	0.040	1.005	0.358										
**Q6**	0.097	0.585	0.039	1.009	0.357										
**Q7**	0.101	0.659	0.033	0.971	0.322										
**Q8**	0.109	0.570	0.042	1.034	0.371										
**Q9**	0.111	0.524	0.040	1.045	0.348										
**Q10**	0.103	0.598	0.042	1.057	0.370										
**Q11**	0.099	0.692	0.033	0.993	0.322										
**Q12**	0.110	0.575	0.039	1.049	0.373										
**Q13**	0.114	0.537	0.038	1.060	0.340										
**Q14**	0.110	0.623	0.039	1.117	0.366										
**Q15**	0.111	0.698	0.037	1.032	0.324										
**Q16**	0.117	0.606	0.038	1.092	0.375										
**Q17**						0.121	0.560	0.042	1.139	0.353					
**Q18**						0.115	0.633	0.047	1.183	0.371					
**Q19**						0.122	0.696	0.046	1.051	0.316					
**Q20**						0.117	0.628	0.044	1.117	0.355					
**Q21**											0.137	0.589	0.037	1.106	0.359
**Q22**											0.123	0.657	0.046	1.096	0.362
**Q23**											0.133	0.759	0.033	1.016	0.336
**Q24**											0.141	0.633	0.044	1.084	0.368

Table 12: Mean predicted likelihood under the assumption that a given complication is positive. Similar to Table S11 above, except that we condition on complications having a positive outcome. This time the highest predicted likelihood per column is expected on the diagonal, which is the case here

Complication	Hyper/hypo-glycemia	Tissue infection	Retinopathy	Cardiovascular events	Amputation
Hyper/hypo-glycemia	0.1985	0.1564	0.0158	0.1298	0.0460
Tissue infection	0.0311	0.1820	0.0099	0.2062	0.0662
Retinopathy	0.0440	0.1312	0.0264	0.1920	0.0603
Cardiovascular events	0.0144	0.1151	0.0083	0.3133	0.0562
Amputation	0.0166	0.1176	0.0072	0.1760	0.0876

.

Supplementary Methods

Comparison with Logistic Regression

In this final section of the Supplementary Material, we compare our model to Logistic Regression, a model commonly used in machine learning for healthcare. However, such a comparison is not trivial. Indeed, our XGBoost model, thanks to the cross-class relevance, can deal with a multi-label target while Logistic Regression cannot. Thus, we train five different Logistic Regression models, one for the adverse outcome prediction of each diabetes complication. We train each model with the same input features as XGBoost. Unlike XGBoost, Logistic Regression needs feature normalization to reach its full potential. We experimented with several normalization techniques, and found that the one leading to the best discrimination was to scale all features to the [0;1] range. Table S7 offers a comparison of discrimination between XGBoost and the Logistic Regression models.

For the three-year buffer analyzed in the main text, we see that XGBoost outperforms Logistic Regression on all tasks, with a gain in AUC between +3.1 (Hyper/hypo-glycemia) and +0.9 (Cardiovascular events), for an average AUC gain of +1.66. While modest, we stress that at the several millions patients scale of our study, a +1-2 AUC point gain could represent costs savings in the order of tens of millions of dollars annually. For a buffer of 1 year, XGBoost also outperforms Logistic Regression on all tasks, with a mean gain of +1.55 AUC point (XGBoost: 81.04, Logistic Regression: 79.49). For a buffer of 5 years, XGBoost outperforms Logistic Regression on all tasks, as well, with a mean gain of +2.09 AUC point (XGBoost: 76.85, Logistic Regression: 74.76).

Supplementary References

1. Gomez-Uribe, C. A. & Hunt, N. The Netflix Recommender System: Algorithms, Business Value, and Innovation. *ACM Trans. Manage. Inf. Syst.* **6**, 1–19 (2016).
2. Covington, P., Adams, J. & Sargin, E. Deep Neural Networks for YouTube Recommendations. in *Proceedings of the 10th ACM Conference on Recommender Systems* 191–198 (Association for Computing Machinery, 2016).
3. Linden, G., Smith, B. & York, J. Amazon.com recommendations: item-to-item collaborative filtering. *IEEE Internet Comput.* **7**, 76–80 (2003).
4. Phelan, O., McCarthy, K. & Smyth, B. Using twitter to recommend real-time topical news. in *Proceedings of the third ACM conference on Recommender systems* 385–388 (Association for Computing Machinery, 2009).
5. Lika, B., Kolomvatsos, K. & Hadjiefthymiades, S. Facing the cold start problem in recommender systems. *Expert Syst. Appl.* **41**, 2065–2073 (2014).
6. Wan-Shiou Yang, Jia-Ben Dia, Hung-Chi Cheng & Hsing-Tzu Lin. Mining Social Networks for Targeted Advertising. in *Proceedings of the 39th Annual Hawaii International Conference on System Sciences (HICSS'06)* vol. 6 137a–137a (2006).
