# Supplementary material for: Predicting adverse outcomes due to diabetes complications with machine learning using administrative health data
Source: NPJ Digit Med. 2021 Feb 12;4:24. doi: 10.1038/s41746-021-00394-8 (PMC7881135; doi:10.1038/s41746-021-00394-8)
Supplement: Supplementary file 2 — Reporting Summary [file 41746_2021_394_MOESM2_ESM.pdf]

nature research

Corresponding author(s): Laura C. Rosella

Last updated by author(s): Jan 4, 2021

Reporting Summary

Nature Research wishes to improve the reproducibility of the work that we publish. This form provides structure for consistency and transparency in reporting. For further information on Nature Research policies, see our Editorial Policies and the Editorial Policy Checklist.

Statistics

For all statistical analyses, confirm that the following items are present in the figure legend, table legend, main text, or Methods section.

n/a	Confirmed	
☐	☒	The exact sample size (*n*) for each experimental group/condition, given as a discrete number and unit of measurement
☐	☒	A statement on whether measurements were taken from distinct samples or whether the same sample was measured repeatedly
☒	☐	The statistical test(s) used AND whether they are one- or two-sided<br>*Only common tests should be described solely by name; describe more complex techniques in the Methods section.*
☐	☒	A description of all covariates tested
☒	☐	A description of any assumptions or corrections, such as tests of normality and adjustment for multiple comparisons
☒	☐	A full description of the statistical parameters including central tendency (e.g. means) or other basic estimates (e.g. regression coefficient) AND variation (e.g. standard deviation) or associated estimates of uncertainty (e.g. confidence intervals)
☒	☐	For null hypothesis testing, the test statistic (e.g. *F*, *t*, *r*) with confidence intervals, effect sizes, degrees of freedom and *P* value noted<br>*Give P values as exact values whenever suitable.*
☒	☐	For Bayesian analysis, information on the choice of priors and Markov chain Monte Carlo settings
☒	☐	For hierarchical and complex designs, identification of the appropriate level for tests and full reporting of outcomes
☒	☐	Estimates of effect sizes (e.g. Cohen's *d*, Pearson's *r*), indicating how they were calculated

Our web collection on statistics for biologists contains articles on many of the points above.

Software and code

Policy information about availability of computer code

Data collection	Data collection was done with custom code by ICES in the SAS Enterprise v6.1 software.
Data analysis	Data analysis was conducted with custom code by Layer 6 AI using the Python 3.6 and Java 8 programming languages.

For manuscripts utilizing custom algorithms or software that are central to the research but not yet described in published literature, software must be made available to editors and reviewers. We strongly encourage code deposition in a community repository (e.g. GitHub). See the Nature Research guidelines for submitting code & software for further information.

Data

Policy information about availability of data

All manuscripts must include a data availability statement. This statement should provide the following information, where applicable:

- Accession codes, unique identifiers, or web links for publicly available datasets
- A list of figures that have associated raw data
- A description of any restrictions on data availability

The dataset for this study is held securely in coded form at ICES. While data sharing agreements prohibit ICES from making the dataset publicly available, access may be granted to those who meet pre-specified criteria for confidential access available at www.ices.on.ca/DAS. The full dataset creation plan and underlying analytic code are available from the authors upon request, understanding that the computer program may rely upon coding templates or macros that are unique to ICES that are therefore either inaccessible or may require modification.

Field-specific reporting

Please select the one below that is the best fit for your research. If you are not sure, read the appropriate sections before making your selection.

☒ Life sciences ☐ Behavioural & social sciences ☐ Ecological, evolutionary & environmental sciences

For a reference copy of the document with all sections, see nature.com/documents/nr-reporting-summary-flat.pdf

Life sciences study design

All studies must disclose on these points even when the disclosure is negative.

Sample size	We used an eleven-year time period from Jan 1, 2006 to Dec 31, 2016 for this study. The Ontario Diabetes Database contained 1,645,089 patients that were flagged as being diagnosed with diabetes at some point in their life and alive on Jan 1, 2006.
Data exclusions	We excluded patients that were not alive as of January 1, 2012 (n=56,345), and immigrant patients who arrived in Canada later than the last test observation window (n=21,108). This resulted in the final cohort of 1,567,636 patients, corresponding to more than 95% of the original cohort.
Replication	All random splitting and machine learning experiments were done with manually chosen random seeds, allowing exact replication of the results.
Randomization	We randomly split the patients into a training set (n= 1,029,366), a validation set (n=272,864), and a test set (n=265,406). Since our goal was to create an accurate, large-scale prediction predictive model using a data-driven approach, we did not control for covariates.
Blinding	We did not need to blind investigators to group allocation since our goal was to make a predictive model with the highest possible performance using a data-driven approach and we did not do a clinical trial.

Reporting for specific materials, systems and methods

We require information from authors about some types of materials, experimental systems and methods used in many studies. Here, indicate whether each material, system or method listed is relevant to your study. If you are not sure if a list item applies to your research, read the appropriate section before selecting a response.

Materials & experimental systems

n/a	Involved in the study	
☒	☐	Antibodies
☒	☐	Eukaryotic cell lines
☒	☐	Palaeontology and archaeology
☒	☐	Animals and other organisms
☐	☒	Human research participants
☒	☐	Clinical data
☒	☐	Dual use research of concern

Methods

n/a	Involved in the study	
☒	☐	ChIP-seq
☒	☐	Flow cytometry
☒	☐	MRI-based neuroimaging

Human research participants

Policy information about studies involving human research participants

Population characteristics	Population characteristics can be found in our Table 1. We did not filter out for specific population characteristics other than the Exclusion Criteria mentioned above.
Recruitment	Participants were recruited from Ontario's comphensive administrative health data holdings at ICES. However, some Indigenous people may be accessing their healthcare through the federal program and these interactions would not be captured in the ICES data.
Ethics oversight	The study protocol was approved by the ICES Privacy and Legal Office and the Research Ethics Board at the University of Toronto (Protocol 37650).

Note that full information on the approval of the study protocol must also be provided in the manuscript.
